# Long-term survey of longhorn beetles revealed changes in faunal features in Ito on the Izu peninsula

**DOI:** 10.1371/journal.pone.0263761

**Published:** 2022-02-18

**Authors:** Junsuke Yamasako, Keizi Kiritani, Hiroshi Makihara, Takehiko Yamanaka

**Affiliations:** 1 Institute for Agro-Environmental Sciences, NARO, Tsukuba, Ibaraki, Japan; 2 1020-292 Futo, Itō, Shizuoka, Japan; 3 Forestry and Forest Products Research Institute, Tsukuba, Ibaraki, Japan; 4 Research Center for Agricultural Information Technology, NARO, Tsukuba, Ibaraki, Japan; Instituto Federal de Educacao Ciencia e Tecnologia Goiano - Campus Urutai, BRAZIL

## Abstract

Long-term biodiversity monitoring is essential for unveiling the impact of environmental changes on local fauna. Although private local records can contribute significantly to biodiversity evaluation, they are seldom published in scientific journals. In this study, a retired scientist recorded the longhorn beetles (Distiniidae and Cerambycidae) present in Ito on the Izu peninsula, Japan, for 12 years. The records showed the dynamical changes in longhorn beetles, which indicated the environmental changes around the survey site over 12 years. We also compared the longhorn beetle composition in the Ito study site to those in the survey records in 13 other locations in Kanto, Japan. We found that the species composition in Ito was stable throughout the 12 years, while the general composition in Ito reflected the land-use pattern of urban areas and the collecting methods. The species composition in the Ito study site differed from that in some of the other *satoyama* locations (human-influenced natural environment), but this was possibly due to methodological differences. Long-term backyard biodiversity surveys, especially those conducted by retired professionals, can play important roles in future investigations of insect groups, such as longhorn beetles, even if they are not agricultural pests nor endangered species.

## Introduction

Anthropogenic activity is causing drastic environmental changes, leading to the current sixth mass extinction on Earth [1]. Long-term biodiversity monitoring is of critical importance to investigate how these anthropogenic impacts affect the local fauna and, conversely, what changes in the local fauna can tell us about environmental degradation [2–4]. Although the long-term monitoring of important agricultural pest insects (e.g. [5, 6]) and iconic conservation priority species, such as butterflies [7], is well established, regular monitoring surveys have seldom been undertaken for other species groups. However, most insect groups, which do not outbreak in agricultural farmlands or are not endangered, may also have important roles in monitoring anthropogenic alterations to the environment.

“Backyard biodiversity surveys” by the public can boost the quantity of long-term survey data for various species [8]. Japan has had large numbers of amateur entomologists for more than 100 years. Longhorn beetles (Insecta, Coleoptera, Distiniidae and Cerambycidae) are among the most popular subjects for Japanese amateur entomologists. Approximately 1,050 species and subspecies of longhorn beetles are known to occur in Japan [9]. Some are forest and orchard pests (e.g. *Anoplophora malasiaca*, *Monochamus alternatus*, and *Psacothea hilaris*); however, the majority of species are non-pest herbivores whose life histories are primarily dependent on the existence of host trees. Thus, the faunal composition of most longhorn beetles is directly influenced by forest conditions [10, 11], and consequently, these species represent important forest bioindicators [12]. Since longhorn beetles are among the most well-studied insect groups [13], there are plenty of existing survey reports (S2 Table). However, these surveys are conducted primarily on a volunteer basis across one season or based on the presence/absence data of museum specimens. To date, there are few monitoring records of longhorn beetles that span more than a decade as fixed-point observations [14, 15].

In our study, one of the authors, KK, who had been a professional entomologist for more than 40 years and retired in 1996 [16], collected data on longhorn beetles every night for 12 years after retirement. We investigated how changes in longhorn beetle communities could indicate changes in the surrounding environment over time. Then, we compared a composition of longhorn beetles in the Ito site to those in 13 other locations in Kanto, Japan to see how land-use patterns or climatic conditions affect the composition of longhorn beetles at the Ito site.

## Methods

### Data collection in Ito and precedent records in Kanto

**Fig 1:**
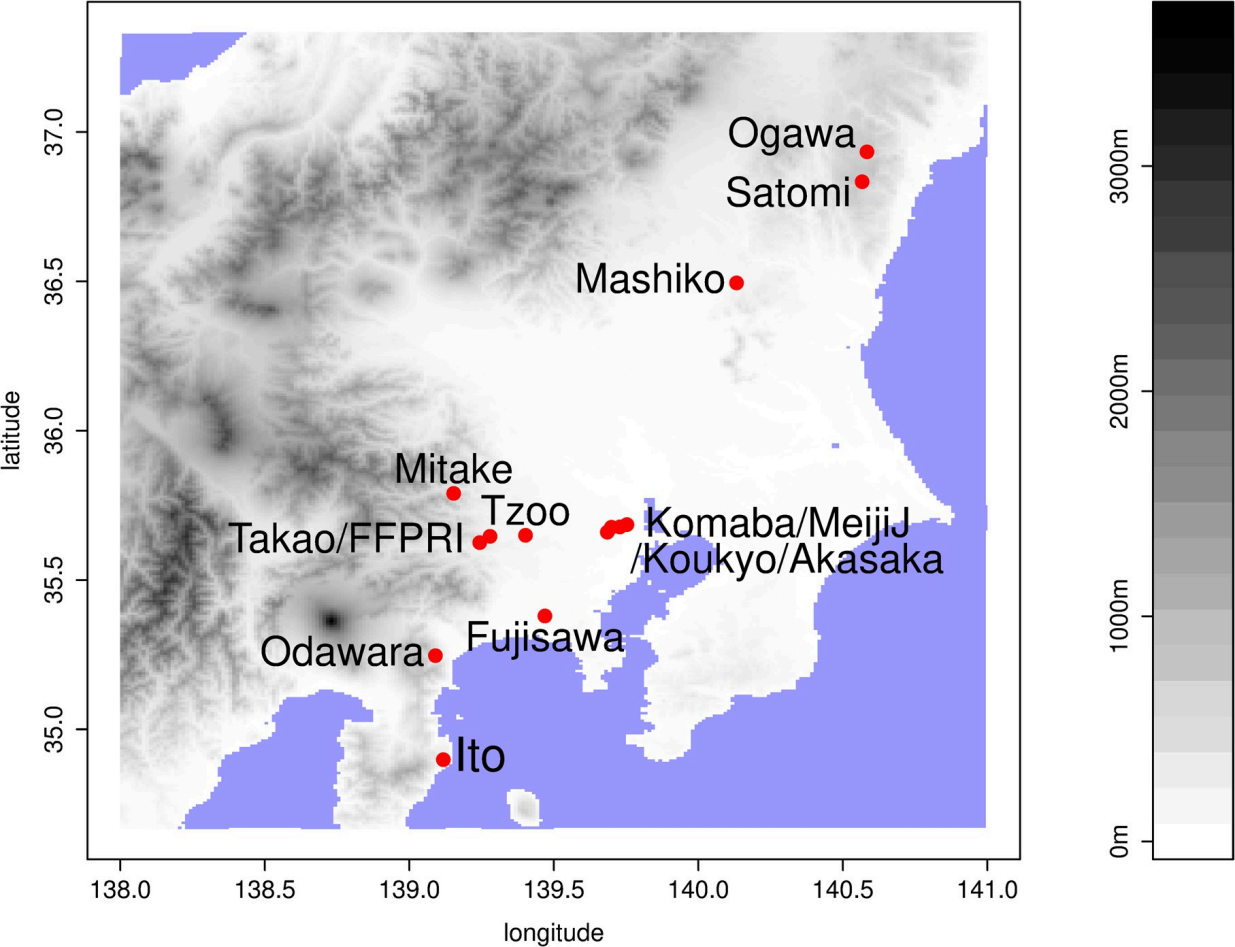
Locations of Ito and the other 13 sampling sites in Kanto. See Table 1 for a detailed description of the sites.

The study region, Ito city, is in the middle of the Izu peninsula (Japan), and was originally known chiefly as a tourist destination; however, in recent years, urban development has progressed (Fig 1). The survey for longhorn beetles was conducted every night (except during severe weather conditions) from mid-May to mid-September for 12 years, from 2004 to 2015. The study site was a residential condominium with 135 units and six floors. Twice each night (21:00–22:00 and 03:00–04:00), the surveyor observed the fluorescent lights in the open corridor and emergency stairs from the first to sixth floors and three outside lights. We assume that the survey covers all nocturnal longhorn beetles, as Baba & Hirashima have mentioned that 20:00 to 2:00 is a suitable period for light-trap monitoring [17].

**Table 1:** Details of the longhorn beetle sampling sites in Kanto.

Location	Source	Study period	Method	Latitude	Longitude	Land use proportion (%) *					Land-use description
						broadL	pine	cedar	agric	resident	
**Ito**	This study	2004–2015	Light	34.90	139.12	33%	8%	16%	14%	12%	satoyama
**Odawara**	Makihara et al. [19]	1987	Attract	35.25	139.09	25%	0%	47%	19%	5%	rural park
**Mashiko**	Makihara et al. [19]	1981–1982	Light	36.49	140.13	33%	12%	7%	33%	5%	satoyama
**Ogawa**	Makino et al. [11]	2003	Malaise	36.93	140.58	31%	4%	33%	23%	0%	satoyama
**Satomi**	Makino et al. [11]	2003	Malaise	36.83	140.57	13%	1%	73%	8%	0%	satoyama
**Akasaka**	Nomura and Hirano [20]	2002–2004	Day	35.68	139.73	1%	0%	0%	0%	97%	urban park
			Malaise								
**Komaba**	Kishimoto-Yamada et al. [21]	2014–2015	Day	35.66	139.69	1%	0%	0%	0%	99%	urban park
			Light								
**Koukyo**	Saito [22]	1996–1998	Day	35.69	139.75	1%	0%	0%	0%	91%	urban park
			Light								
			Attract								
**MeijiJ**	Okada et al. [23]	2011–2012	Day	35.68	139.70	1%	0%	0%	0%	99%	urban park
			Light								
			Malaise								
**Mitake**	Yagishita [24]	1988–2006	Day	35.79	139.15	12%	0%	83%	0%	5%	Mountainous area
			Light								
**Fujisawa**	Takano [25]	2009–2013	Day	35.38	139.47	5%	0%	7%	20%	67%	urban park
**Tzoo**	Sakurai et al. [26]	1963–2008	Day	35.65	139.40	16%	0%	1%	5%	67%	rural park
			Light								
**FFPRI**	Matsumoto et al. [27]	1989–1991, 2001–2012	Day	35.65	139.28	20%	0%	31%	5%	43%	rural park
			Attract								
**Takao**	Fujita [28]	1932–1986	Day	35.63	139.24	20%	0%	53%	5%	17%	Mountainous area
			Light								

* Land-use abbreviations. broadL: broad leaf woodlots; pine: pine tree forest; cedar: cedar tree plantations; agric: crop fields and paddies; resident: residential area.

N.B., land-use proportions were calculated using the 3rd mesh land-use information provided by the Biodiversity Center of Japan (http://www.biodic.go.jp/dload/mesh_vg.html, accessed on 4 Oct 2020).

To contrast the communities of longhorn beetles in Ito with others, we compared the total number of each longhorn beetle species at our study site with those of previous surveys of longhorn beetles in 13 other locations in the Kanto region, Japan (Table 1). Like Ito, the environment in Ogawa, Satomi, and Mashiko comprises a mostly *satoyama* landscape (i.e. a human-influenced natural environment, for definition, see Takeuchi [18]) with pine trees and broad-leaved tree forests, and a few urban areas. Mitake and Takao are in a mountainous area (>500m in altitude). Considering their surrounding environments, Odawara, Tama Zoo (Tzoo), and the Garden of Forestry and Forest Products Institute (FFPRI) are categorized as rural parks. Akasaka, Komaba, Kouyo, and Meiji Jungu (MeijiJ) are urban parks in the middle of the metropolitan area in Tokyo. Fujisawa is also an urban park in a suburban residential area. As these data were mostly presence/absence records, our Ito data were also converted into the presence/absence format in this comparison.

### Meteorological records in Ito

As for meteorological factors in the Ito site, we calculated the mean temperatures and the total amount of precipitation in the fall (from August to November in the last year), winter (from December of the last year to March of the year) and spring (from April to July of the year) for each year. Daily records of the temperature and precipitation were taken from the nearest AMeDAS (Automated Meteorological Data Acquisition System) weather station, Ajiro, 11 km away from Ito station (https://www.jma.go.jp/jma/en/Activities/amedas/amedas.html, accessed on 12 Jul 2021).

### Landuse and meteorological records in Kanto

For the analysis of the 14 Kanto sites including Ito, land-use information was retrieved from the Biodiversity Center of Japan (www.biodic.go.jp, accessed on 4 Oct 2020). The land-use information around our 14 census stations was extracted from the fourth national vegetation surveys (1988–1992) for Odawara, Mashiko, Mitake, Fujisawa, Tzoo, FFPRI and Takao and fifth surveys (1992–1996) for Ito, Ogawa, Satomi, Akasaka, Komaba, Kouyo, and MeijiJ depending on each study period (Table 1). The vegetation types were recorded in the national third grid resolution (ca. 1 km × 1 km), and all data within a 5-km buffer radius surrounding the sites (considering the dispersal ability of longhorn beetles, e.g., see [29]) were pooled. The land-use data were further categorised into 1) areas occupied by broad-leaved trees (broadL); 2) areas of *Pinus densiflora* and *P*. *thunbergii* (pine); 3) plantations of *Cryptomeria japonica* and *Chamaecyparis obtusa* (cedar); 4) agricultural crop fields (agric); 5) residential areas (resident); 6) water surfaces, golf courses (others). The relative proportions (%) of these categories were used as land-use factors in the ordination analysis of the 14 Kanto sites, which is described later in the next section.

A mean monthly temperature and precipitation value for each station from 1981 to 2010 were also downloaded from National Land Numerical Information (https://nlftp.mlit.go.jp/ksj/, accessed on 4 June 2021) for the 14 Kanto analysis. Because there are only 14 stations in our study and many predictors cannot be considered in our ordination analysis, a principal component analysis (PCA) was introduced to summarize meteorological factors into a few representative predictors. The mean monthly temperatures and precipitation values, i.e. 24 variables in total in the 14 stations, were incorporated into the PCA. Two primary axes were used for further analyses since they explained 95% of the variations of the original 24 variables. To enhance the interpretability of the PCA axes, a varimax rotation was employed after PCA (Fig 3D) [30]. The first axis (PCA1, named meteo1) generally explained temperature effects while the secondary axis (PCA2, as meteo2) explained precipitation effects; both were incorporated into the ordination analysis, that is described later in the next section, as climatic factors.

### Ordination analyses in Ito and Kanto

We employed a direct ordination, redundancy analysis (RDA), to characterize the temporal changes in the community of longhorn beetles in Ito based upon the linear time trend and the meteorological factors [31, 32]. As our longhorn beetle data in Ito for each year was highly skewed, the number of captures in each species was transformed using a Hellinger transformation (square root of a capture of a species divided by the total captures in each year) [33]. The significance of the linear time trend (year itself) and the meteorological factors (e.g. mean seasonal temperatures and total precipitation) were tested by the forward and backward stepwise parameter selection. The stepwise procedure starts from a null model (a simple principal component analysis without predictors) and adding and removing each factor one-by-one. Then, the selection and rejection of the factors are decided according to an ANOVA-like permutation test of 999 iterations [34]. *Ad-hoc* round-robin tests based on Kendall’s correlation coefficient were conducted between the factors and the numbers of each species.

Another RDA was also applied to the total occurrence of longhorn beetles in 14 Kanto sites including Ito in Table 1. Presence/absence data were directly incorporated into the RDA analysis without Hellinger transformation. The influences of land use and meteorological factors on longhorn beetle communities were tested in the RDA analyses. In addition, the effect of collecting methods was also incorporated in the RDA analysis. The surveys in 14 stations were conducted either by a light-trap census (Light), daytime collections (Day) such as beating net, sweeping net, and visual inspections, Malaise trap (Malaise), and/or an attraction trap (Attract), with attraction chemicals such as benzyl-acetate, Madara-call®, Akane-call®, and/or Kogane-call® (Sankei Chemical Co., Ltd., Kagoshima, Japan). Collection methods were coded in the RDA as dummy variables.

Instead of evaluating all the predictors by the ANOVA-like test, we eliminated nauseant predictors causing multicollinearity by the variance inflation factors (VIF) analysis for each factor group, land use, climate, or method, respectively [35]. If there are multiple VIFs larger than 10 (the threshold of multicollinearity), we dropped the factor having the lowest adjusted R^2^ value for the RDA, sequentially, until all the VIFs get to the values smaller than 10. After removing nauseant predictors using VIF analysis, we employed variation partitioning to quantify the relative contributions of the three factor groups: land use, climate, and method [36, 37]. That is, we firstly conducted RDAs of climate, land use, and method, respectively. We, then, conducted an additional RDA, including predictors of all the three factor groups simultaneously and those of the two factor group pairs. Then, the valuations of the pure components, the paired components, and the full suite of the three factor groups were calculated. The relative contributions of the pure and overlapped effects were determined based on the variations of the sole and the combined RDAs.

To see the relationship between the number of species in each station and its relative position in the RDA ordination space, a smooth surface of the number of species was overlaid onto the RDA ordination space using a generalised additive model [38] of the smooth term *s*(RDA1, RDA2), where *s*() is a spline-based smoothing function controlled by a fixed parameter-*k* (*k* = 10).

In addition, *ad-hoc* plots of the 12-year data from Ito were also overlaid onto the ordination space with the 14 Kanto sites to visualize the dynamical changes of the composition of longhorn beetles in Ito in conjunction with the faunal variation of longhorn beetles in the Kanto region. Ito’s 12 years records were converted into the presence/absence format.

The *vegan* library was employed for the RDA analyses, and the overlays were performed by a generalised additive model using the *gam* library [39, 40].

## Results

### Longhorn beetle community at Ito

**Fig 2:**
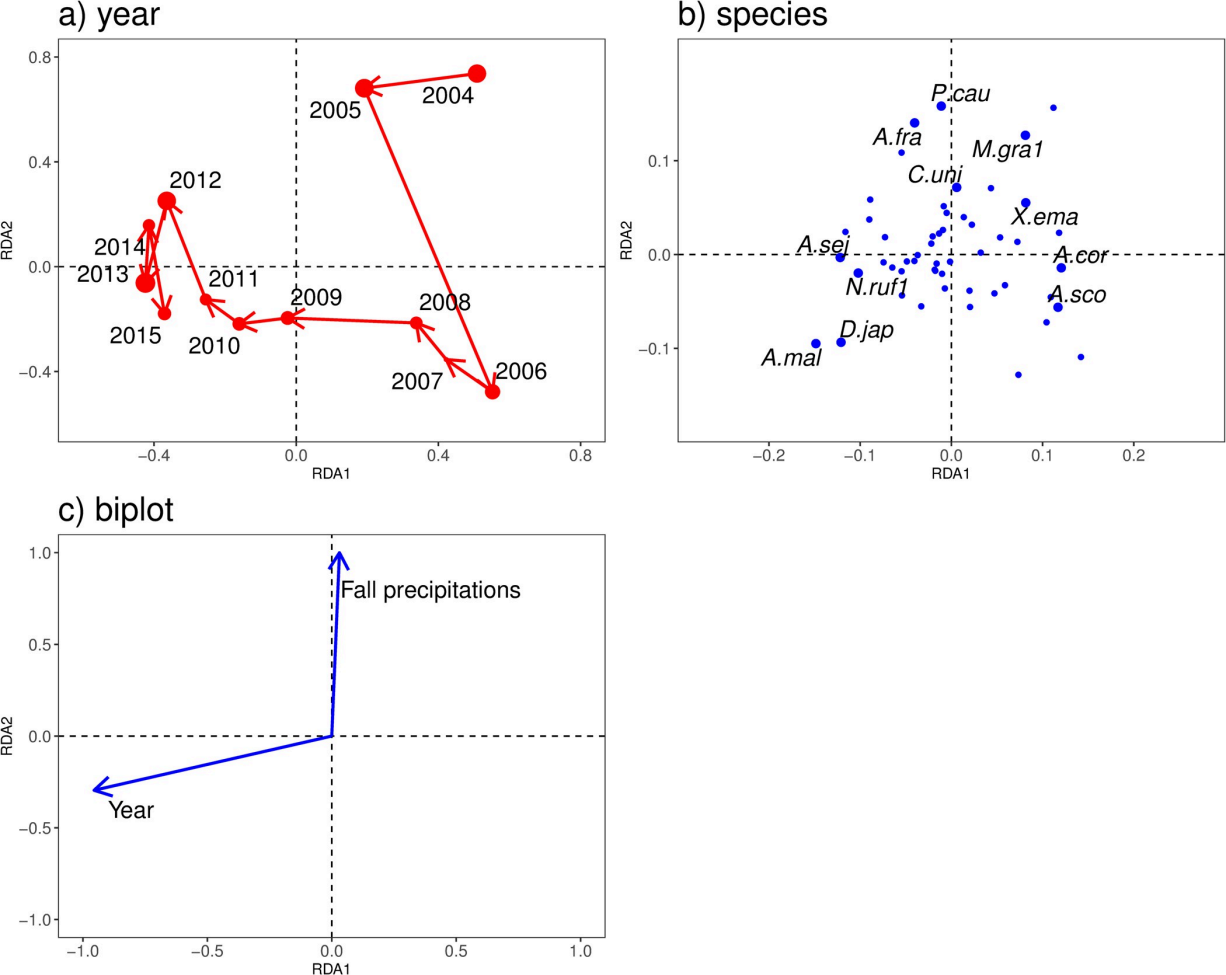
RDA ordination diagram of the species composition of longhorn beetles (Cerambycidae and Disteniidae) at Ito city (a: plot by year, b: species plot, c: biplot). The size of the circles in panel a represents the total number of longhorn beetles captured each year (e.g. 44 individuals in 2007 and 173 in 2013). See S1 Table for the species codes in panel b. Only those having statistically significant correlations to Year and Fall precipitations and *A*.*mal* are displayed in panel b.

In total, 58 species and 1,276 longhorn beetles were captured over the 12-year study period (S1 Table). From RDA, only the yearly trend (*p* < 0.001 in ANOVA-like permutation test with null model) and the fall precipitation in the last year (*p* < 0.03) were selected by the stepwise parameter selection (Fig 2C), and no other parameters (i.e., the spring and winter precipitations, spring, fall, and winter temperatures) were selected. The biplot of the yearly data indicated faunal changes in longhorn beetles in Ito among years (Fig 2A). *A*. *malasiaca* (*A*.*mal*), *Acalolepta sejuncta* (*A*.*sej*), *Distenia japonica* (*D*.*jap*), and *Nysina rufescens* (*N*.*ruf1*), were plotted in the direction of the yearly increasing trend, while *Arhopalus coreanus* (*A*.*cor*), *Anastrangalia scotodes* (*A*.*sco*), and *Xylotrechus emaciatus* (*X*.*ema*) were located along the yearly decreasing trends (Fig 2B). Round-robin tests of Kendall’s correlation were significantly positive for *D*. *japonica* (*D*.*jap*, *p* = 0.03), *A*. *sejuncta* (*A*.*sej*, *p* = 0.05), and *N*. *rufescens* (*N*.*ruf1*, *p* = 0.02) and significantly negative for *A*. *coreanus* (*A*.*cor*), *A*. *scotodes* (*A*.*sco*), and *X*. *emaciatus* (*X*.*ema*). *A*. *malasiaca* (*A*.*mal*), which was located the furthest left and downward in the ordination space, was not marginally significant in relation to the increasing trend (*p* = 0.06).

The total amount of fall precipitation in the last year had a positive effect towards the upper part of the ordination space (Fig 2A). *Pterolophia caudata* (*P*.*cau*), *Acalolepta fraudator* (*A*.*fra*), *Molorchus gracilis* (*M*.*gra1*), and *Cephalallus unicolor* (*C*.*uni*) were located in the positive direction of the fall precipitation having significant positive correlations (Kendall’s test) with the fall precipitation (*p* = 0.03, *p* = 0.04, *p* = 0.01, and *p* = 0.004, respectively), while no negative correlations were detected.

The number of individuals captured ranged from 44 (2007) to 173 (2013) and showed no consistent pattern with yearly trends (*p* = 0.638) nor ordination axes (*p* = 0.947 for RDA1 and *p* = 0.250 for RDA2) based on Kendall’s rank correlation test.

### Comparison of longhorn beetle communities in Ito and other Kanto sites

**Fig 3:**
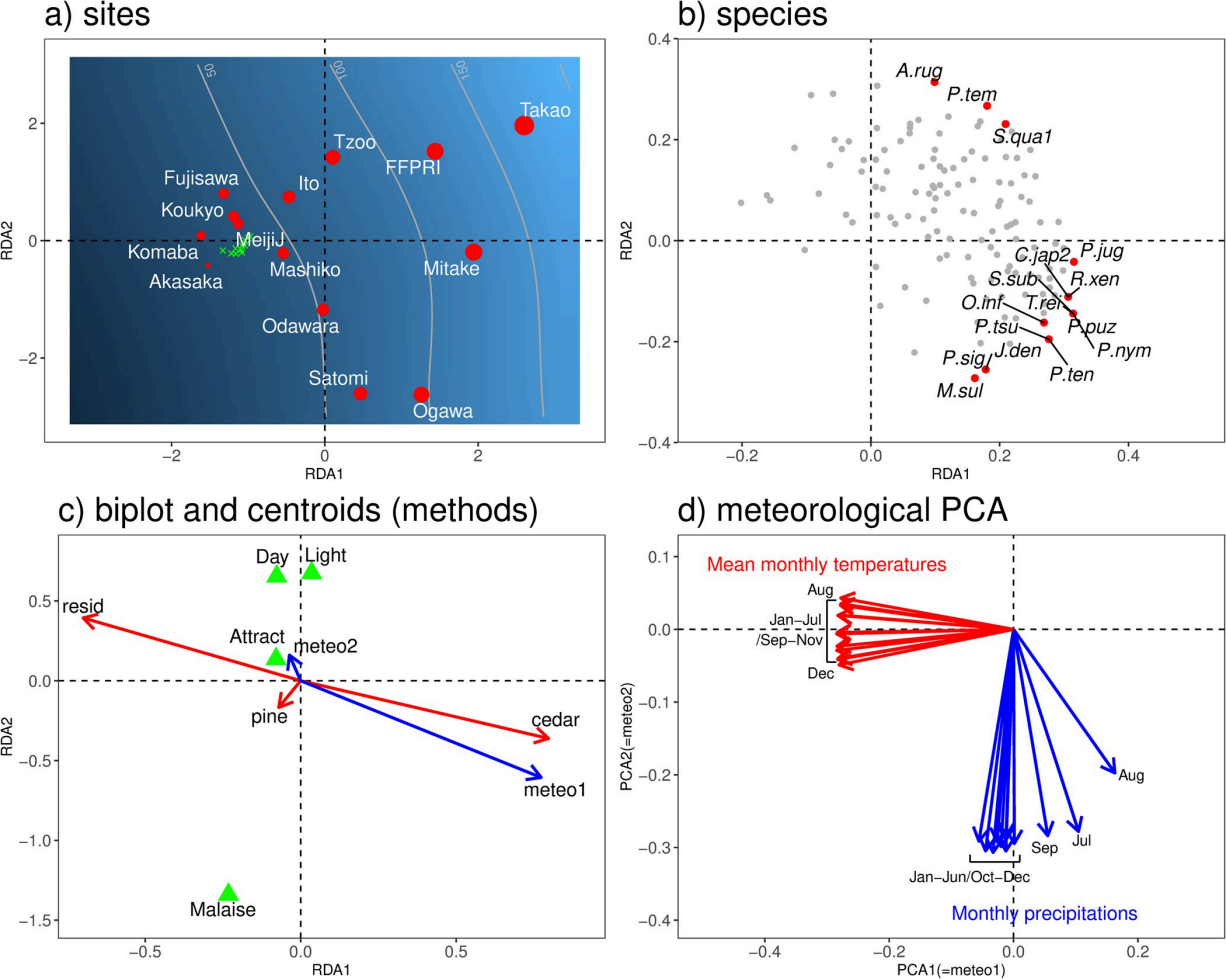
RDA ordination diagram of species composition of longhorn beetles across the 14 study sites in the Kanto region (a: plot by sites, b: species plot, c: biplot and centroids of factors), and biplot of climatic PCA (d). Total number of species were overlayed as contour, gradient, and the size of the points (a brighter gradient and a bigger point indicate a greater number of species) in panel a. *Ad-hoc* plots of the 12-year data from Ito were also overlaid onto panel a (green crosses). Only 16 species, whose locations are furthest from the ordination centre, are shown in panel b (See S2 Table for abbreviations of species). Factors are plotted as arrows, but the methods (Day, Light, Attract, and Malaise) are as the centroids of the stations in panel c. meteo1 and meteo2 in RDA are the coordinates of stations in the metrological PCA of panel d.

RDA of longhorn beetle communities in Kanto revealed a monotonic gradation along RDA1 from low species richness on the left side to high species richness on the right side (Fig 3A). This trend was confirmed by the *gam* model, *s*(RDA1, RDA2), overlaid onto the ordination space (*p* < 0.0001). A simple Kendall rank correlation between RDA1 and the numbers of species was also highly significant (*p* = 0.003).

Using the variance inflation factor analysis, we eliminated agr and broadL from the land use RDA while we use all variables for climate and method. Generally, warmer stations were located on the left of the ordination space, while cooler stations were on the right (Fig 3C and 3D). Urban parks, such as Komaba, Akasaka, Koukyo, and MeijiJ were located on the left, where the temperature was warm, and the diversity of longhorn beetles was low (Fig 3A). Rural parks and mountainous areas, such as Mitake, Tzoo, FFPRI, and Takao, were located on the right side of the ordination space, where the temperature was cool and the species richness high (Fig 3A). Ito, Mashiko, Ogawa, and Satomi, i.e., the *satoyama* sites, were located in the middle, between urban parks and mountainous area (Fig 3A). The climatic gradient condition corresponded to the land use patterns, i.e., cedar plantation increased with the cooler climatic sites in mountain areas and residential areas prevailed in warmer urban areas.

*Ad-hoc* plots of the 12-year species composition in Ito were overlaid onto the ordination space (green crosses in Fig 3A). They moved toward the lower left from the original position because the varieties of species in each year were poorer than those summed for the 12 years. The 12-year data formed a small cluster to the middle left, where species richness was poor. This indicates that the variety of species did not change during the 12 years in comparison to the longhorn beetle composition in Kanto.

Many flower beetles, such as *Pidonia signifera* (*P*.*sig*), *Japanostrangalia dentatipennis* (*J*.*den*), *Parastrangalis tenuicornis* (*P*.*ten*), *Toxotinus reinii* (*T*.*rei*), *Parastrangalis nymphula* (*P*.*nym*), *Pidonia puziloi* (*P*.*puz*), *Menesia sulphurata* (*M*.*sul*), and *Oberea infranigrescens* (*O*.*inf*), were located in the lower right of the ordination space, which corresponded to cedar plantations (cedar) or a cooler climate (meteo1) (Fig 3B). *Apriona rugicollis* (*A*.*rug*), *Purpuricenus temminckii* (*P*.*tem*), and *Schwarzerium quadricolle* (*S*.*qua1*) were located at the upper right-hand side of the ordination space.

**Fig 4:**
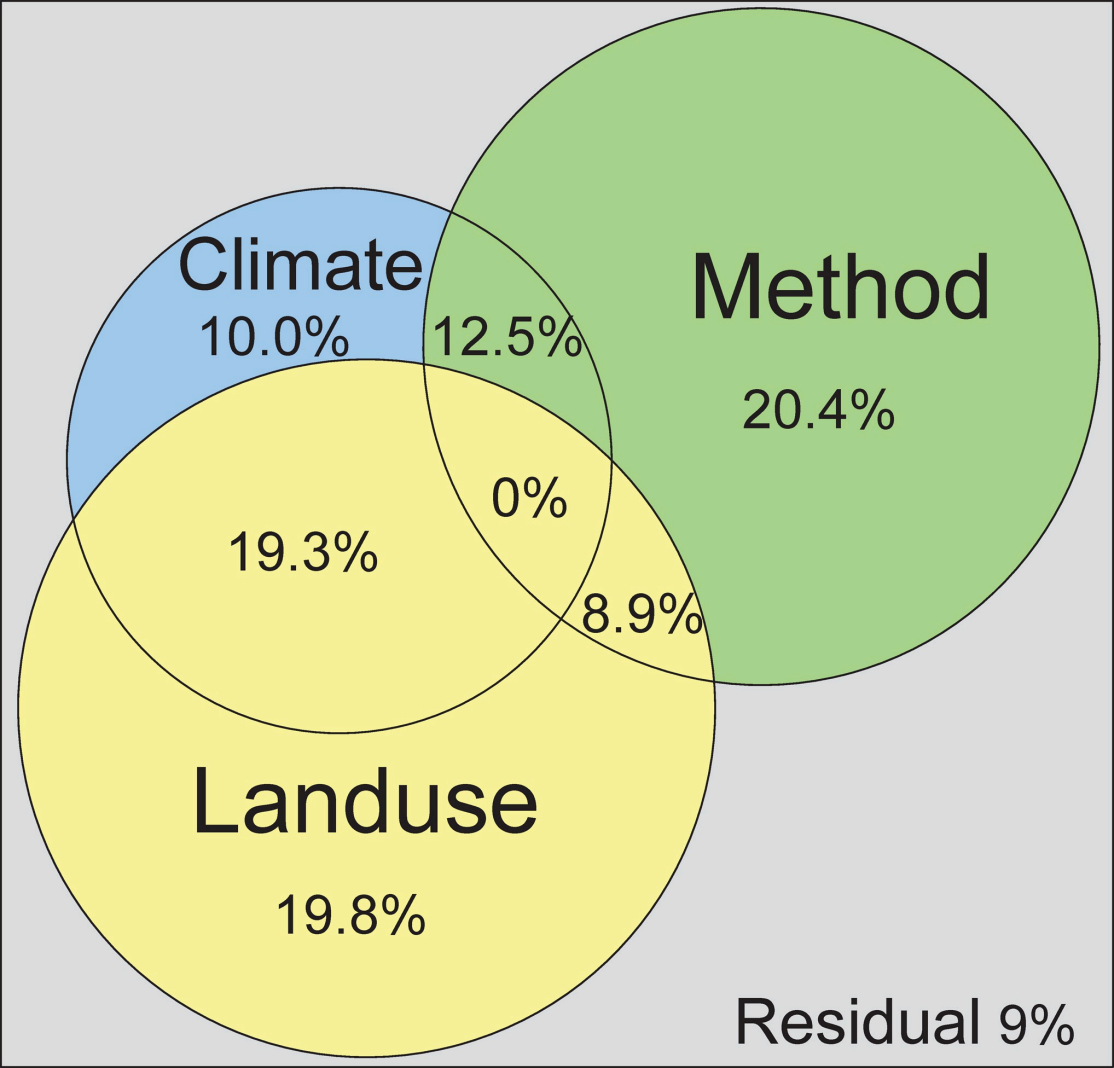
Variation partitioning among the three factor groups of climate, land use, and method. The sizes of the circles roughly represent the relative strength of the effects on the community structure of the longhorn beetles in Kanto.

Variation partitioning revealed that land use and method made substantial and nearly equal contributions to the longhorn beetles’ community variation while climate had the minimal contribution among the three (Fig 4). Land use and climate together contributed 19.3% to the variation in the longhorn beetle composition, while land use and method contributed 8.9% and climate and method contributed 12.5%.

## Discussion

Generally, the abundance and species composition of longhorn beetle communities are strongly affected by the surrounding environment, especially tree composition (e.g., [10, 11]). Though we could not directly compare the dynamical changes in tree composition around the Ito site with our longhorn beetles’ community, the increasing and declining trends observed in the abundance of longhorn beetles at the survey site over the 12 years of observation is likely to indicate changes in the surrounding environment rather than merely yearly fluctuations. The species located on the left side, i.e., yearly increasing trends in the RDA ordination space (Fig 2B), generally have high tolerance to urbanisation. On the other hand, typical *satoyama* species decreased. Especially, *A*. *coreanus* (*A*.*cor*) and *A*. *scotodes* (*A*.*sco*) are pine feeders and were on the right side of the ordination species (Fig 2B). The significant decrease in the species related to pine trees might reflect the degradation of pine tree forest due to pine wilt disease that has expanded into the Kanto area since the 1980s [41]. We suspect that these temporal trends in the longhorn beetle composition in Ito indicate that the surrounding environment changed from a rural *satoyama* environment with rich pine woodlots to an urbanised resort area.

We also found that *P*. *caudata* (*P*.*cau*), *A*. *fraudator* (*A*.*fra*), *M*. *gracilis* (*M*.*gra1*), and *C*. *unicolor* (*C*.*uni*) were significantly positively correlated with fall precipitation (Fig 2). Because these species are generally uni-voltine and emerge as adults in the summer, the fall precipitation in the last year may influence the survival of their young larvae. Indeed, several studies on other longhorn beetles have reported that fall precipitation positively affects larval survival (e.g. [42, 43]). Although we still do not know why the presence of only these four species correlated with the fall precipitation while the other uni-voltine species did not in our study, we conclude that the rainfall in the previous year had a major effect on the longhorn beetle community.

It should be noted that the community structure in Ito drastically changed in 2006, and the number of captures was generally low from 2007 to 2011. We suspect that there were unexpected events such as the development of residential land, deforestation in the surrounding environment, and/or methodological differences between the periods before 2006 and after 2007. Although we did not record the types of lights in our Ito station, some fluorescent lights were replaced with LEDs (especially outside lights) during our study period. As LEDs usually produce highly monochromatic light, they sometimes repel nocturnal insects rather than collecting them [44].

The RDA of the 14 Kanto stations revealed that the longhorn beetle communities were mainly influenced by the land use around the study sites as well as the collecting method. Indeed, 19.8% of the variation in the longhorn beetle community was solely explained by land use (48.0% in total). The relationship is simple, i.e., cooler, mountainous areas with a rich forest cover (cedar plantations) had a greater variety of longhorn beetles than other habitats (Fig 3). The collecting method contributed 20.4% to the community structure second to land use (41.8% in total). Satomi and Ogawa are located at the bottom of the RDA ordination space (Fig 3A), this corresponds to the effect of the Malaise trap (Fig 3C).

Although we found a dynamical change in the community of longhorn beetles in the 12-year analysis in Ito, the species composition was stable throughout the period in the Kanto analysis (Fig 3A). Since we employed Hellinger transformation in the Ito analysis, the relative proportion of the abundance of every species was decomposed in the ordination space, while presence/absence combinations were used in the Kanto analysis. Therefore, we could have detected the gradual trend from a typical *satoyama* species composition to that in an urbanised environment in Ito. However, the species numbers did not drastically change considering the result of the Kanto analysis. Though the compositional change in longhorn beetle communities in time in Ito was significant in the single site analysis, it was not conspicuous in the Kanto analysis. We suspect that environmental constraints, that is, land use and climatic conditions, influenced the longhorn beetle community at the regional scale.

The total composition of the longhorn beetles in Ito for the 12-year period was similar to that of the other *satoyama* location, Mashiko. In the ordination space, these locations were located at an intermediate position between mountainous areas and urbanised parks. Although mountainous, forested areas are the most important habitats for longhorn beetles in terms of conservation priority, *satoyama* locations can also host some species. Satomi and Ogawa are also classified as *satoyama* but were located slightly differently as compared to Ito and Mashiko in the ordination space (Fig 3A). We suspect that this was due to methodological differences as was mentioned above. Ordinally, we cannot compare community structures among studies with different methods. However, our variation partitioning successfully separated methodological influences from land use and climate effects.

It should be noted that our 14 study sites in Kanto were the records from different years (1932–2015) with various survey durations (from one year to 55 years) (Table 1). Actually, sites with longer survey durations and older records had tendencies of having larger numbers of species than shorter and more recent surveys by simple correlation analyses (results not shown). However, the survey sites of old and long durations such as Takao, Mitake, FFPRI, and Tzoo are located in the mountain and cool areas while the sites of new and short survey durations, such as in Komaba, Akasaka, Koukyo, and MeijiJ are located in the urban and warm areas. In addition, one-year surveys in Satomi, Ogawa and Odawara could have collected more species than the five years survey in Fujisawa. This may be caused not only by environmental difference but also by the methodological difference, i.e., the Malaise and attraction traps used in Satomi, Ogawa and Odawara could have effectively collected diverse species than daytime catching in Fujisawa or reflect the actual faunal differences between the sites. We could not segregate the effect of the year and the duration from the other factors, and this is the limitation of this study.

We evaluated environmental changes and faunal features in Ito from a 12-year-long private observational survey of longhorn beetles by a retired professional scientist. Personal observations are usually listed in local club magazines for naturalists and seldom published in scientific journals because surveys like these are typically conducted in a short period at a single location. Moreover, data quality can usually not be guaranteed in such personal observations. However, retired professional scientists can use their professional knowledge and experience in collecting and identifying insects. We envision that long-term backyard biodiversity surveys, especially those conducted by retired professionals, can play an important role in future investigations of insect groups, such as longhorn beetles, even if these species are not agricultural pests nor endangered.

## Supporting information

S1 Table Full list of the Ito survey. (DOCX)

S2 Table List of Kanto longhorn census locations (presence/absence data). (DOCX)
