# Supplementary material for: Long-term survey of longhorn beetles revealed changes in faunal features in Ito on the Izu peninsula
Source: PLoS One. 2022 Feb 18;17(2):e0263761. doi: 10.1371/journal.pone.0263761 (PMC8856520; doi:10.1371/journal.pone.0263761)
Supplement: S1 Table — (DOCX) [file pone.0263761.s001.docx]

**S1 Table** Full list of the Ito survey

| **Scientific name** | **Short name** | **2004** | **2005** | **2006** | **2007** | **2008** | **2009** | **2010** | **2011** | **2012** | **2013** | **2014** | **2015** |
| --- | --- | --- | --- | --- | --- | --- | --- | --- | --- | --- | --- | --- | --- |
| *Acalolepta* (*Acalolepta*) *fraudator fraudator* (Bates) | *A.fra* | 24 | 37 | 8 | 3 | 9 | 9 | 5 | 17 | 23 | 16 | 13 | 10 |
| *Acalolepta* (*Acalolepta*) *luxuriosa luxuriosa* (Bates) | *A.lux* | 4 | 1 | 3 | 1 | 2 | 2 | 3 | 1 | 2 | 8 | 3 | 3 |
| *Acalolepta* (*Acalolepta*) *sejuncta sejuncta* (Bates) | *A.sej* | 0 | 0 | 0 | 0 | 0 | 0 | 0 | 2 | 4 | 3 | 0 | 1 |
| *Aegosoma sinicum sinicum* White | *A.sin* | 3 | 5 | 4 | 3 | 6 | 4 | 1 | 3 | 5 | 6 | 3 | 3 |
| *Allotraeus sphaerioninus* Bates | *A.sph* | 0 | 0 | 0 | 0 | 0 | 0 | 0 | 0 | 0 | 0 | 1 | 0 |
| *Anastrangalia scotodes scotodes* (Bates) | *A.sco* | 0 | 1 | 2 | 1 | 1 | 0 | 0 | 0 | 0 | 0 | 0 | 0 |
| *Anoplophora malasiaca* (Thomson) | *A.mal* | 5 | 14 | 11 | 4 | 7 | 20 | 21 | 12 | 17 | 27 | 13 | 17 |
| *Apriona* (*Apriona*) *rugicollis rugicollis* Chevrolat | *A.rug* | 1 | 1 | 1 | 1 | 2 | 0 | 0 | 1 | 0 | 1 | 0 | 0 |
| *Arhopalus coreanus* (Sharp) | *A.cor* | 4 | 0 | 2 | 0 | 2 | 0 | 0 | 0 | 0 | 0 | 0 | 0 |
| *Batocera lineolata* Chevrolat | *B.lin* | 2 | 1 | 2 | 0 | 2 | 0 | 3 | 2 | 5 | 3 | 0 | 0 |
| *Cephalallus unicolor unicolor* (*Gahan*) | *C.uni* | 5 | 7 | 1 | 1 | 1 | 3 | 7 | 2 | 5 | 2 | 2 | 1 |
| *Chlorophorus muscosus* (Bates) | *C.mus* | 2 | 1 | 0 | 0 | 1 | 0 | 1 | 0 | 1 | 0 | 0 | 0 |
| *Chlorophorus quinquefasciatus* (Castelnau & Gory) | *C.qui* | 0 | 0 | 0 | 0 | 0 | 0 | 1 | 1 | 0 | 0 | 0 | 0 |
| *Distenia* (*Distenia*) *japonica japonica* Bates | *D.jap* | 1 | 0 | 1 | 0 | 3 | 10 | 2 | 4 | 2 | 4 | 3 | 4 |
| *Eurypoda* (*Neoprion*) *batesi* Gahan | *E.bat* | 0 | 0 | 0 | 0 | 0 | 0 | 1 | 0 | 0 | 0 | 0 | 0 |
| *Eutetrapha ocelota* (Bates) | *E.oce* | 0 | 0 | 0 | 0 | 0 | 0 | 1 | 0 | 1 | 0 | 0 | 0 |
| *Exocentrus guttulatus guttulatus* Bates | *E.gut* | 0 | 0 | 0 | 0 | 0 | 0 | 1 | 0 | 1 | 7 | 0 | 0 |
| *Exocentrus lineatus* Bates | *E.lin* | 0 | 0 | 0 | 0 | 0 | 0 | 1 | 0 | 0 | 1 | 0 | 0 |
| *Falsomesosella* (*Falsomesosella*) *gracilior* (Bates) | *F.gra* | 0 | 0 | 0 | 0 | 0 | 0 | 0 | 0 | 0 | 0 | 1 | 0 |
| *Grammographus notabilis notabilis* (Pascoe) | *G.not* | 0 | 0 | 2 | 0 | 0 | 0 | 0 | 0 | 0 | 0 | 0 | 0 |
| *Leptura* (*Leptura*) *ochraceofasciata ochraceofasciata* (Motschulsky) | *L.och* | 1 | 0 | 5 | 1 | 1 | 2 | 1 | 0 | 0 | 1 | 0 | 0 |
| *Leptura* (*Noona*) *regalis* (Bates) | *L.reg* | 0 | 0 | 0 | 0 | 0 | 0 | 1 | 0 | 0 | 0 | 0 | 0 |
| *Margites* (*Margites*) *fulvidus* (Pascoe) | *M.ful* | 0 | 0 | 0 | 0 | 0 | 0 | 0 | 0 | 0 | 0 | 0 | 1 |
| *Mecynippus pubicornis* Bates | *M.pub* | 0 | 1 | 0 | 0 | 0 | 0 | 0 | 0 | 0 | 0 | 0 | 0 |
| *Mesosa* (*Aplocnemia*) *longipennis* Bates | *M.lon* | 2 | 0 | 8 | 1 | 3 | 3 | 1 | 1 | 4 | 6 | 0 | 1 |
| *Mesosa* (*Mesosa*) *japonica* Bates | *M.jap* | 0 | 0 | 0 | 0 | 0 | 0 | 0 | 0 | 0 | 0 | 0 | 1 |
| *Mesosa* (*Perimesosa*) *hirsuta hirsuta* Bates | *M.hir* | 0 | 0 | 0 | 0 | 0 | 0 | 0 | 0 | 0 | 1 | 0 | 0 |

**S1 Table** (continued1)

| **Scientific name** | **Short name** | **2004** | **2005** | **2006** | **2007** | **2008** | **2009** | **2010** | **2011** | **2012** | **2013** | **2014** | **2015** | |
| --- | --- | --- | --- | --- | --- | --- | --- | --- | --- | --- | --- | --- | --- | --- |
| *Mimectatina meridiana ohirai* Breuning & Villiers | *M.mer* | 0 | 0 | 0 | 0 | 0 | 0 | 0 | 0 | 1 | 0 | 0 | 0 |  |
| *Monochamus* (*Monochamus*) *alternatus endai* Makihara | *M.alt* | 2 | 0 | 0 | 1 | 0 | 1 | 0 | 0 | 0 | 0 | 0 | 0 | |
| *Monochamus* (*Monochamus*) *grandis* Waterhouse | *M.gra1* | 7 | 2 | 0 | 0 | 0 | 1 | 2 | 0 | 0 | 0 | 0 | 0 | |
| *Monochamus* (*Monochamus*) *subfasciatus* *subfasciatus* Bates | *M.sub* | 10 | 6 | 14 | 1 | 2 | 0 | 3 | 0 | 3 | 1 | 3 | 2 | |
| *Niphona* (*Niphona*) *furcata* (Bates) | *N.fur* | 0 | 0 | 1 | 0 | 0 | 0 | 0 | 0 | 0 | 0 | 0 | 1 | |
| *Nysina rufescens rufescens* (Pic) | *N.ruf1* | 0 | 0 | 0 | 0 | 0 | 0 | 0 | 1 | 1 | 4 | 0 | 1 | |
| *Oberea* (*Oberea*) *hebescens* Bates | *O.heb* | 0 | 0 | 0 | 0 | 0 | 0 | 1 | 0 | 0 | 0 | 0 | 0 | |
| *Palimna liturata liturata* (Bates) | *P.lit* | 0 | 0 | 0 | 1 | 0 | 0 | 0 | 0 | 0 | 1 | 0 | 0 | |
| *Paraglenea fortunei* (Saunders) | *P.for* | 0 | 1 | 1 | 0 | 0 | 0 | 1 | 0 | 0 | 0 | 1 | 2 | |
| *Pareutetrapha simulans* (Bates) | *P.sim1* | 1 | 2 | 1 | 2 | 0 | 1 | 0 | 0 | 1 | 1 | 0 | 1 | |
| *Prionus insularis insularis* Motschulsky | *P.ins* | 8 | 12 | 11 | 6 | 11 | 12 | 12 | 7 | 10 | 6 | 4 | 4 | |
| *Psacothea hilaris hilaris* (Pascoe) | *P.hil* | 7 | 9 | 3 | 3 | 6 | 6 | 4 | 9 | 13 | 11 | 2 | 13 | |
| *Psephactus remiger remiger* Harold | *P.rem* | 0 | 1 | 0 | 0 | 0 | 0 | 1 | 0 | 1 | 0 | 0 | 0 | |
| *Pseudaeolesthes chrysothrix chrysothrix* (Bates) | *P.chr1* | 7 | 10 | 5 | 8 | 9 | 3 | 5 | 3 | 16 | 15 | 7 | 12 | |
| *Pterolophia* (*Hylobrotus*) *annulata* (Chevrolat) | *P.ann* | 0 | 1 | 0 | 0 | 0 | 0 | 0 | 0 | 0 | 2 | 0 | 0 | |
| *Pterolophia* (*Pterolophia*) *caudata caudata* (Bates) | *P.cau* | 12 | 9 | 1 | 0 | 1 | 1 | 1 | 0 | 2 | 7 | 4 | 1 | |
| *Pterolophia* (*Pterolophia*) *granulata* (Motschulsky) | *P.gra1* | 0 | 1 | 0 | 0 | 0 | 0 | 1 | 0 | 2 | 5 | 1 | 0 | |
| *Pterolophia* (*Pterolophia*) *leiopodina* (Bates) | *P.lei* | 1 | 0 | 0 | 0 | 0 | 0 | 0 | 0 | 0 | 0 | 0 | 0 | |
| *Pterolophia* (*Pterolophia*) *zonata* (Bates) | *P.zon* | 2 | 1 | 0 | 0 | 0 | 0 | 0 | 0 | 7 | 8 | 0 | 0 | |
| *Purpuricenus temminckii temminckii* Guerin-Meneville | *P.tem* | 2 | 0 | 0 | 0 | 0 | 0 | 0 | 0 | 0 | 0 | 1 | 0 | |
| *Rhodopina lewisii lewisii* (Bates) | *R.lew* | 0 | 1 | 0 | 0 | 0 | 1 | 1 | 0 | 5 | 2 | 1 | 0 | |
| *Rhopaloscelis unifasciata* Blessig | *R.uni* | 0 | 0 | 0 | 0 | 0 | 0 | 0 | 0 | 0 | 1 | 0 | 0 | |
| *Rondibilis* (*Rondibilis*) *saperdina* (Bates) | *R.sap* | 1 | 0 | 0 | 0 | 0 | 1 | 0 | 0 | 0 | 0 | 0 | 1 | |
| *Rosalia batesi* Harold | *R.bat* | 0 | 0 | 0 | 0 | 0 | 0 | 0 | 0 | 1 | 0 | 2 | 0 | |
| *Spondylis buprestoides* (Linnaeus) | *S.bup* | 29 | 18 | 2 | 3 | 8 | 5 | 0 | 5 | 7 | 5 | 2 | 4 | |
| *Stenhomalus* (*Stenhomalus*) *cleroides* Bates | *S.cle* | 0 | 0 | 0 | 0 | 0 | 1 | 0 | 0 | 0 | 2 | 0 | 0 | |
| *Uraecha bimaculata bimaculata* Thomson | *U.bim* | 5 | 11 | 17 | 3 | 3 | 3 | 4 | 1 | 9 | 13 | 6 | 2 | |

**S1 Table** (continued2)

| **Scientific name** | **Short name** | **2004** | **2005** | **2006** | **2007** | **2008** | **2009** | **2010** | **2011** | **2012** | **2013** | **2014** | **2015** | |
| --- | --- | --- | --- | --- | --- | --- | --- | --- | --- | --- | --- | --- | --- | --- |
| *Xenicotela pardalina* (Bates, 1884) | *X.par* | 0 | 0 | 0 | 0 | 0 | 0 | 0 | 0 | 4 | 2 | 2 | 0 |  |
| *Xylotrechus* (*Xylotrechus*) *cuneipennis* (Kraatz, 1879) | *X.cun* | 0 | 0 | 0 | 0 | 0 | 0 | 1 | 0 | 0 | 1 | 1 | 0 | |
| *Xylotrechus* (*Xylotrechus*) *emaciatus* Bates, 1884 | *X.ema* | 1 | 2 | 1 | 0 | 0 | 2 | 0 | 0 | 0 | 0 | 0 | 0 | |
| *Xystrocera globosa* (Olivier, 1795) | *X.glo* | 1 | 0 | 0 | 0 | 0 | 0 | 0 | 0 | 0 | 0 | 0 | 0 | |
