# Supplementary material for: Long-term survey of longhorn beetles revealed changes in faunal features in Ito on the Izu peninsula
Source: PLoS One. 2022 Feb 18;17(2):e0263761. doi: 10.1371/journal.pone.0263761 (PMC8856520; doi:10.1371/journal.pone.0263761)
Supplement: S2 Table — (DOCX) [file pone.0263761.s002.docx]

**S2 Table** List of Kanto longhorn census locations (presence/absence data)

| **Scientific name** | **Short name** | **Ito** | **Ogawa** | **Satomi** | **Mashiko** | **Odawara** | **Komaba** | **Koukyo** | **Akasaka** | **FFPRI** | **Fujisawa** | **Takao** | **MeijiJ** | **Mitake** | **Tzoo** |
| --- | --- | --- | --- | --- | --- | --- | --- | --- | --- | --- | --- | --- | --- | --- | --- |
| *Acalolepta* (*Acalolepta*) *fraudator fraudator* (Bates) | *A.fra* | 1 | 1 | 1 | 1 | 1 | 0 | 1 | 0 | 1 | 0 | 1 | 1 | 1 | 1 |
| *Acalolepta* (*Acalolepta*) *kusamai* Hayashi | *A.kus* | 0 | 0 | 0 | 0 | 0 | 0 | 1 | 1 | 1 | 0 | 1 | 0 | 0 | 0 |
| *Acalolepta* (*Acalolepta*) *luxuriosa luxuriosa* (Bates) | *A.lux* | 1 | 0 | 0 | 0 | 1 | 1 | 0 | 0 | 1 | 1 | 1 | 1 | 1 | 1 |
| *Acalolepta* (*Acalolepta*) *sejuncta sejuncta* (Bates) | *A.sej* | 1 | 1 | 1 | 1 | 1 | 0 | 1 | 0 | 1 | 0 | 1 | 1 | 1 | 0 |
| *Acanthocinus* (*Acanthobatesianus*) *guttatus* (Bates) | *A.gut* | 0 | 0 | 0 | 1 | 0 | 0 | 0 | 0 | 0 | 0 | 1 | 0 | 0 | 1 |
| *Acanthocinus* (*Acanthocinus*) *orientalis* K. Ohbayashi | *A.ori* | 0 | 0 | 0 | 1 | 0 | 0 | 0 | 0 | 1 | 0 | 1 | 0 | 0 | 1 |
| *Aegosoma sinicum sinicum* White | *A.sin* | 1 | 0 | 0 | 0 | 0 | 1 | 1 | 1 | 1 | 1 | 1 | 1 | 1 | 1 |
| *Allotraeus sphaerioninus* Bates | *A.sph* | 1 | 0 | 0 | 0 | 1 | 0 | 0 | 0 | 1 | 0 | 1 | 0 | 1 | 0 |
| *Anaglyptus* (*Akajimatora*) *bellus* Matsumura & Matsushita | *A.bel* | 0 | 0 | 0 | 0 | 0 | 0 | 0 | 0 | 1 | 0 | 1 | 0 | 1 | 0 |
| *Anaglyptus* (*Anaglyptus*) *matsushitai* Hayashi | *A.mat* | 0 | 0 | 0 | 0 | 0 | 0 | 0 | 0 | 0 | 0 | 1 | 0 | 1 | 0 |
| *Anaglyptus* (*Anaglyptus*) *niponensis* Bates | *A.nip1* | 0 | 0 | 0 | 0 | 1 | 0 | 1 | 1 | 1 | 0 | 1 | 1 | 1 | 0 |
| *Anaglyptus* (*Anaglyptus*) *subfasciatus* Pic | *A.sub* | 0 | 0 | 0 | 0 | 1 | 0 | 0 | 0 | 0 | 0 | 0 | 0 | 0 | 0 |
| *Anastrangalia scotodes scotodes* (Bates) | *A.sco* | 1 | 1 | 1 | 1 | 1 | 0 | 0 | 0 | 1 | 0 | 1 | 0 | 1 | 0 |
| *Anoploderomorpha excavata* (Bates) | *A.exc* | 0 | 1 | 1 | 0 | 0 | 0 | 0 | 0 | 0 | 0 | 1 | 0 | 1 | 0 |
| *Anoplophora malasiaca* (Thomson) | *A.mal* | 1 | 1 | 0 | 1 | 0 | 0 | 0 | 0 | 1 | 1 | 1 | 0 | 0 | 1 |
| *Apomecyna naevia naevia* Bates | *A.nae* | 0 | 0 | 0 | 0 | 0 | 1 | 1 | 0 | 0 | 0 | 1 | 1 | 0 | 1 |
| *Apriona* (*Apriona*) *rugicollis rugicollis* Chevrolat | *A.rug* | 1 | 0 | 0 | 0 | 0 | 0 | 1 | 0 | 1 | 1 | 1 | 1 | 1 | 1 |
| *Arhopaloscelis nipponensis* (Pic) | *A.nip2* | 0 | 1 | 0 | 0 | 1 | 0 | 1 | 0 | 1 | 0 | 1 | 1 | 1 | 0 |
| *Arhopalus coreanus* (Sharp) | *A.cor* | 1 | 0 | 0 | 1 | 0 | 0 | 0 | 0 | 1 | 0 | 1 | 0 | 1 | 1 |
| *Asaperda agapanthina* Bates | *A.aga* | 0 | 1 | 1 | 1 | 1 | 0 | 1 | 1 | 1 | 1 | 1 | 1 | 1 | 1 |
| *Asaperda rufipes* Bates | *A.ruf* | 0 | 1 | 0 | 1 | 0 | 0 | 0 | 0 | 1 | 0 | 1 | 0 | 0 | 0 |
| *Asemum striatum* (Linnaeus) | *A.str* | 0 | 0 | 0 | 1 | 0 | 0 | 0 | 0 | 1 | 0 | 1 | 0 | 0 | 0 |
| *Astynoscelis degener* (Bates) | *A.deg* | 0 | 0 | 0 | 0 | 0 | 0 | 0 | 0 | 0 | 0 | 1 | 0 | 0 | 0 |
| *Atimura japonica* Bates | *A.jap* | 0 | 1 | 0 | 0 | 0 | 0 | 1 | 0 | 0 | 0 | 1 | 0 | 1 | 0 |
| *Aulaconotus pachypezoides* Thomson | *A.pac* | 0 | 0 | 0 | 0 | 0 | 0 | 0 | 0 | 1 | 1 | 1 | 0 | 0 | 0 |

**S2 Table** (continued 1)

| **Scientific name** | **Short name** | **Ito** | **Ogawa** | **Satomi** | **Mashiko** | **Odawara** | **Komaba** | **Koukyo** | **Akasaka** | **FFPRI** | **Fujisawa** | **Takao** | **MeijiJ** | **Mitake** | **Tzoo** |
| --- | --- | --- | --- | --- | --- | --- | --- | --- | --- | --- | --- | --- | --- | --- | --- |
| *Bacchisa* (*Bacchisa*) *fortunei japonica* (Gahan) | *B.for* | 0 | 0 | 0 | 0 | 0 | 1 | 0 | 0 | 0 | 0 | 1 | 0 | 0 | 1 |
| *Batocera lineolata* Chevrolat | *B.lin* | 1 | 0 | 0 | 0 | 0 | 0 | 0 | 0 | 1 | 0 | 1 | 0 | 1 | 1 |
| *Brachyclytus singularis* Kraatz | *B.sin* | 0 | 1 | 0 | 0 | 1 | 0 | 0 | 0 | 0 | 0 | 1 | 0 | 0 | 0 |
| *Cagosima sanguinolenta* Thomson | *C.san* | 0 | 0 | 0 | 0 | 0 | 0 | 0 | 0 | 1 | 0 | 1 | 0 | 0 | 1 |
| *Callapoecus guttatus* Bates | *C.gut* | 0 | 0 | 0 | 0 | 0 | 0 | 0 | 0 | 0 | 0 | 0 | 0 | 1 | 0 |
| *Callidiellum rufipenne* (Motschulsky) | *C.ruf* | 0 | 0 | 1 | 0 | 1 | 1 | 1 | 0 | 1 | 1 | 1 | 0 | 1 | 1 |
| *Cephalallus unicolor unicolor* (Gahan) | *C.uni* | 1 | 0 | 0 | 0 | 1 | 0 | 0 | 0 | 1 | 0 | 1 | 0 | 1 | 0 |
| *Ceresium sinicum sinicum* White | *C.sin* | 0 | 0 | 0 | 0 | 0 | 1 | 1 | 1 | 1 | 0 | 0 | 1 | 0 | 0 |
| *Chloridolum* (*Leontium*) *viride* (Thomson) | *C.vir* | 0 | 0 | 0 | 0 | 0 | 0 | 0 | 0 | 1 | 0 | 1 | 0 | 0 | 1 |
| *Chloridolum* (*Parachloridolum*) *japonicum* (Harold) | *C.jap1* | 0 | 0 | 0 | 0 | 0 | 0 | 0 | 0 | 0 | 0 | 0 | 0 | 0 | 1 |
| *Chlorophorus annularis* (Fabricius) | *C.ann* | 0 | 0 | 0 | 0 | 0 | 0 | 0 | 0 | 0 | 0 | 1 | 0 | 0 | 1 |
| *Chlorophorus diadema inhirsutus* Matsushita | *C.dia* | 0 | 0 | 0 | 1 | 0 | 0 | 0 | 0 | 0 | 0 | 1 | 0 | 0 | 0 |
| *Chlorophorus japonicus* (Chevrolat) | *C.jap2* | 0 | 1 | 1 | 1 | 1 | 0 | 0 | 0 | 1 | 0 | 1 | 0 | 1 | 1 |
| *Chlorophorus muscosus* (Bates) | *C.mus* | 1 | 0 | 0 | 0 | 0 | 0 | 0 | 0 | 0 | 0 | 0 | 0 | 0 | 0 |
| *Chlorophorus quinquefasciatus* (Castelnau & Gory) | *C.qui* | 1 | 0 | 0 | 0 | 0 | 1 | 0 | 0 | 0 | 1 | 0 | 0 | 0 | 0 |
| *Cleptometopus bimaculatus* (Bates) | *C.bim* | 0 | 1 | 1 | 0 | 0 | 0 | 0 | 0 | 0 | 0 | 1 | 0 | 1 | 1 |
| *Clytus* (*Clytus*) *auripilis* Bates | *C.aur* | 0 | 1 | 0 | 0 | 0 | 0 | 0 | 0 | 1 | 0 | 1 | 0 | 0 | 0 |
| *Clytus* (*Clytus*) *melaenus* Bates | *C.mel* | 0 | 1 | 0 | 0 | 0 | 0 | 0 | 0 | 0 | 0 | 1 | 0 | 0 | 0 |
| *Corennys sericata* Bates | *C.ser* | 0 | 0 | 0 | 0 | 0 | 0 | 0 | 0 | 0 | 0 | 0 | 0 | 1 | 0 |
| *Corymbia succedanea* (Lewis, 1879) | *C.suc* | 0 | 0 | 0 | 0 | 0 | 0 | 0 | 0 | 1 | 0 | 1 | 1 | 1 | 1 |
| *Cylindilla grisescens* Bates | *C.gri* | 0 | 0 | 0 | 0 | 0 | 0 | 0 | 0 | 0 | 0 | 0 | 0 | 1 | 0 |
| *Cyrtoclytus caproides caproides* (Bates) | *C.cap* | 0 | 1 | 1 | 1 | 0 | 0 | 0 | 0 | 1 | 0 | 1 | 0 | 1 | 1 |
| *Demonax transilis* Bates | *D.tra* | 0 | 1 | 1 | 1 | 1 | 0 | 1 | 0 | 1 | 1 | 1 | 1 | 1 | 1 |
| *Dere thoracica* White | *D.tho* | 0 | 0 | 0 | 1 | 0 | 0 | 0 | 0 | 1 | 0 | 1 | 0 | 0 | 1 |
| *Dinoptera minuta criocerina* (Bates) | *D.min* | 0 | 1 | 1 | 0 | 0 | 1 | 1 | 1 | 1 | 1 | 1 | 1 | 1 | 1 |
| *Distenia* (*Distenia*) *japonica japonica* Bates | *D.jap* | 1 | 1 | 1 | 1 | 1 | 0 | 1 | 0 | 1 | 0 | 1 | 0 | 1 | 1 |
| *Dolichoprosopus yokoyamai* (Gressitt) | *D.yok* | 0 | 1 | 0 | 0 | 0 | 0 | 0 | 0 | 0 | 0 | 1 | 0 | 0 | 0 |
| *Egesina* (*Niijimaia*) *bifasciana bifasciana* (Matsushita) | *E.bif* | 0 | 0 | 1 | 0 | 0 | 1 | 0 | 0 | 0 | 1 | 1 | 1 | 1 | 1 |

**S2 Table** (continued 2)

| **Scientific name** | **Short name** | **Ito** | **Ogawa** | **Satomi** | **Mashiko** | **Odawara** | **Komaba** | **Koukyo** | **Akasaka** | **FFPRI** | **Fujisawa** | **Takao** | **MeijiJ** | **Mitake** | **Tzoo** |
| --- | --- | --- | --- | --- | --- | --- | --- | --- | --- | --- | --- | --- | --- | --- | --- |
| *Enoploderes* (*Pyrenoploderes*) *bicolor* K. Ohbayashi | *E.bic* | 0 | 0 | 0 | 0 | 0 | 0 | 0 | 0 | 0 | 0 | 1 | 0 | 0 | 0 |
| *Epiclytus yokoyamai* (Kano) | *E.yok* | 0 | 1 | 0 | 0 | 0 | 0 | 0 | 0 | 0 | 0 | 1 | 0 | 1 | 1 |
| *Epiglenea comes comes* Bates | *E.com* | 0 | 0 | 0 | 1 | 1 | 0 | 0 | 0 | 1 | 0 | 1 | 1 | 1 | 1 |
| *Etorofus* (*Nakanea*) *vicarius* (Bates) | *E.vic* | 0 | 1 | 0 | 0 | 0 | 0 | 0 | 0 | 0 | 0 | 0 | 0 | 1 | 0 |
| *Eumecocera argyrosticta* (Bates) | *E.arg* | 0 | 0 | 1 | 0 | 0 | 0 | 0 | 0 | 0 | 0 | 0 | 0 | 0 | 0 |
| *Eumecocera gleneoides* (Gressitt) | *E.gle* | 0 | 1 | 1 | 0 | 0 | 0 | 0 | 0 | 0 | 0 | 1 | 0 | 0 | 0 |
| *Eumecocera trivittata* (Breuning) | *E.tri* | 0 | 1 | 0 | 0 | 0 | 0 | 0 | 0 | 1 | 0 | 1 | 0 | 0 | 0 |
| *Eupogoniopsis tenuicornis* (Bates) | *E.ten* | 0 | 0 | 0 | 0 | 0 | 0 | 0 | 0 | 0 | 0 | 1 | 0 | 1 | 0 |
| *Eupromus ruber* (Dalman) | *E.rub* | 0 | 0 | 0 | 0 | 0 | 0 | 1 | 0 | 0 | 1 | 0 | 0 | 0 | 0 |
| *Eurypoda* (*Neoprion*) *batesi* Gahan | *E.bat* | 1 | 0 | 0 | 0 | 0 | 0 | 0 | 0 | 0 | 0 | 0 | 0 | 0 | 0 |
| *Eustrangalis distenioides* Bates | *E.dis* | 0 | 1 | 0 | 0 | 0 | 0 | 0 | 0 | 0 | 0 | 0 | 0 | 0 | 0 |
| *Eutetrapha chrysochloris chrysochloris* (Bates) | *E.chr* | 0 | 1 | 0 | 0 | 0 | 0 | 0 | 0 | 0 | 0 | 0 | 0 | 0 | 0 |
| *Eutetrapha ocelota* (Bates) | *E.oce* | 1 | 0 | 0 | 1 | 0 | 0 | 1 | 0 | 1 | 0 | 1 | 1 | 1 | 1 |
| *Exocentrus fasciolatus fasciolatus* Bates | *E.fas* | 0 | 0 | 0 | 1 | 0 | 1 | 1 | 0 | 0 | 1 | 1 | 1 | 0 | 1 |
| *Exocentrus galloisi* Matsushita | *E.gal* | 0 | 0 | 0 | 0 | 0 | 0 | 1 | 0 | 1 | 0 | 1 | 1 | 1 | 1 |
| *Exocentrus guttulatus guttulatus* Bates | *E.gut* | 1 | 0 | 0 | 0 | 0 | 0 | 1 | 0 | 1 | 0 | 1 | 0 | 0 | 1 |
| *Exocentrus lineatus* Bates | *E.lin* | 1 | 1 | 0 | 1 | 0 | 1 | 0 | 0 | 1 | 1 | 1 | 1 | 0 | 1 |
| *Exocentrus testudineus* Matsushita | *E.tes* | 0 | 1 | 1 | 1 | 0 | 0 | 1 | 0 | 1 | 0 | 1 | 0 | 1 | 0 |
| *Falsomesosella* (*Falsomesosella*) *gracilior* (Bates) | *F.gra* | 1 | 1 | 0 | 0 | 0 | 0 | 0 | 0 | 1 | 0 | 1 | 0 | 0 | 0 |
| *Glenea* (*Glenea*) *centroguttata* Fairmaire | *G.cen* | 0 | 0 | 0 | 0 | 0 | 0 | 0 | 0 | 0 | 1 | 0 | 0 | 0 | 0 |
| *Glenea* (*Glenea*) *relicta relicta* Pascoe | *G.rel* | 0 | 1 | 1 | 1 | 1 | 0 | 0 | 0 | 1 | 0 | 1 | 1 | 1 | 1 |
| *Grammographus notabilis notabilis* (Pascoe) | *G.not* | 1 | 1 | 0 | 1 | 0 | 0 | 0 | 0 | 1 | 1 | 1 | 1 | 1 | 1 |
| *Graphidessa venata venata* Bates | *G.ven* | 0 | 1 | 1 | 0 | 0 | 0 | 0 | 0 | 1 | 0 | 0 | 0 | 1 | 0 |
| *Idiostrangalia contracta* (Bates) | *I.con* | 0 | 0 | 0 | 0 | 0 | 0 | 0 | 0 | 1 | 0 | 1 | 0 | 1 | 0 |
| *Idiostrangalia hakonensis* (Matsushita) | *I.hak* | 0 | 0 | 0 | 0 | 0 | 0 | 0 | 0 | 0 | 0 | 0 | 0 | 1 | 0 |
| *Japanocorus caeruleipennis* (Bates) | *J.cae* | 0 | 0 | 0 | 0 | 0 | 0 | 0 | 0 | 1 | 0 | 1 | 0 | 1 | 0 |
| *Japanomesosa poecila* (Bates) | *J.poe* | 0 | 1 | 0 | 0 | 0 | 0 | 0 | 0 | 0 | 0 | 0 | 0 | 0 | 0 |
| *Japanostrangalia dentatipennis* (Pic) | *J.den* | 0 | 1 | 1 | 0 | 1 | 0 | 0 | 0 | 0 | 0 | 0 | 0 | 1 | 0 |

**S2 Table** (continued 3)

| **Scientific name** | **Short name** | **Ito** | **Ogawa** | **Satomi** | **Mashiko** | **Odawara** | **Komaba** | **Koukyo** | **Akasaka** | **FFPRI** | **Fujisawa** | **Takao** | **MeijiJ** | **Mitake** | **Tzoo** |
| --- | --- | --- | --- | --- | --- | --- | --- | --- | --- | --- | --- | --- | --- | --- | --- |
| *Judolia japonica* (Tamanuki) | *J.jap* | 0 | 1 | 0 | 0 | 0 | 0 | 0 | 0 | 0 | 0 | 1 | 0 | 0 | 0 |
| *Judolidia bangi bangi* (Pic) | *J.ban* | 0 | 1 | 1 | 0 | 0 | 0 | 0 | 0 | 0 | 0 | 0 | 0 | 1 | 0 |
| *Kanekoa azumensis* (Matsushita & Tamanuki) | *K.azu* | 0 | 0 | 0 | 0 | 0 | 0 | 0 | 0 | 0 | 0 | 0 | 0 | 1 | 0 |
| *Kazuoclytus lautoides* (Hayashi) | *K.lau* | 0 | 0 | 0 | 0 | 0 | 0 | 0 | 0 | 0 | 0 | 1 | 0 | 0 | 0 |
| *Konoa granulata* (Bates) | *K.gra* | 0 | 0 | 0 | 0 | 0 | 0 | 0 | 0 | 0 | 0 | 1 | 0 | 0 | 0 |
| *Leiopus* (*Leiopus*) *stillatus* (Bates) | *L.sti* | 0 | 1 | 0 | 0 | 0 | 0 | 0 | 0 | 1 | 0 | 1 | 0 | 0 | 0 |
| *Lemula* (*Lemula*) *decipiens* Bates | *L.dec* | 0 | 1 | 0 | 0 | 1 | 0 | 0 | 0 | 1 | 0 | 1 | 0 | 1 | 0 |
| *Lemula* (*Lemula*) *nishimurai* Seki | *L.nis* | 0 | 1 | 0 | 0 | 0 | 0 | 0 | 0 | 0 | 0 | 0 | 0 | 0 | 0 |
| *Lemula* (*Lemula*) *rufithorax* Pic | *L.ruf* | 0 | 1 | 0 | 0 | 0 | 0 | 0 | 0 | 0 | 0 | 0 | 0 | 1 | 0 |
| *Leptostrangalia hosohana* (K. Ohbayashi) | *L.hos* | 0 | 0 | 0 | 0 | 0 | 0 | 0 | 0 | 0 | 0 | 0 | 0 | 1 | 0 |
| *Leptura* (*Leptura*) *annularis mimica* Bates | *L.mim* | 0 | 0 | 0 | 0 | 0 | 0 | 0 | 0 | 0 | 0 | 1 | 0 | 0 | 0 |
| *Leptura* (*Leptura*) *annularis modicenotata* Pic | *L.mod* | 0 | 1 | 1 | 0 | 1 | 0 | 0 | 0 | 1 | 0 | 0 | 0 | 1 | 1 |
| *Leptura* (*Leptura*) *dimorpha* Bates | *L.dim* | 0 | 0 | 0 | 0 | 1 | 0 | 0 | 0 | 1 | 0 | 1 | 0 | 1 | 1 |
| *Leptura* (*Leptura*) *kusamai kusamai* K. Ohbayashi & Nakane | *L.kus* | 0 | 0 | 0 | 0 | 0 | 0 | 0 | 0 | 0 | 0 | 0 | 0 | 1 | 0 |
| *Leptura* (*Leptura*) *latipennis* (Matsushita) | *L.lat* | 0 | 1 | 0 | 0 | 0 | 0 | 0 | 0 | 0 | 0 | 0 | 0 | 0 | 0 |
| *Leptura* (*Leptura*) *ochraceofasciata ochraceofasciata* (Motschulsky) | *L.och* | 1 | 1 | 1 | 1 | 1 | 0 | 0 | 0 | 1 | 0 | 1 | 0 | 1 | 1 |
| *Leptura* (*Noona*) *regalis* (Bates) | *L.reg* | 1 | 0 | 1 | 0 | 0 | 0 | 0 | 0 | 1 | 0 | 1 | 0 | 1 | 0 |
| *Margites* (*Margites*) *fulvidus* (Pascoe) | *M.ful* | 1 | 0 | 0 | 0 | 0 | 0 | 0 | 0 | 0 | 0 | 1 | 0 | 0 | 0 |
| *Mecynippus pubicornis* Bates | *M.pub* | 1 | 0 | 0 | 0 | 0 | 0 | 0 | 0 | 0 | 0 | 1 | 0 | 0 | 1 |
| *Megasemum quadricostulatum* Kraatz | *M.qua* | 0 | 1 | 1 | 0 | 0 | 0 | 0 | 0 | 0 | 0 | 1 | 0 | 1 | 0 |
| *Menesia flavotecta* Heyden | *M.fla* | 0 | 0 | 0 | 0 | 0 | 0 | 0 | 0 | 1 | 0 | 1 | 0 | 0 | 0 |
| *Menesia sulphurata* (Gebler) | *M.sul* | 0 | 1 | 1 | 1 | 1 | 0 | 0 | 0 | 0 | 0 | 0 | 0 | 1 | 0 |
| *Mesosa* (*Aplocnemia*) *longipennis* Bates | *M.lon* | 1 | 1 | 0 | 1 | 0 | 1 | 1 | 0 | 1 | 1 | 1 | 1 | 1 | 1 |
| *Mesosa* (*Aplocnemia*) *senilis* Bates | *M.sen* | 0 | 1 | 0 | 0 | 0 | 0 | 0 | 0 | 0 | 0 | 1 | 0 | 1 | 0 |
| *Mesosa* (*Mesosa*) *japonica* Bates | *M.jap* | 1 | 1 | 1 | 0 | 0 | 0 | 0 | 0 | 1 | 0 | 1 | 0 | 1 | 1 |
| *Mesosa* (*Mesosa*) *mediofasciata* Breuning | *M.med* | 0 | 0 | 0 | 0 | 0 | 0 | 0 | 0 | 1 | 0 | 1 | 0 | 0 | 0 |
| *Mesosa* (*Perimesosa*) *hirsuta hirsuta* Bates | *M.hir* | 1 | 0 | 1 | 1 | 0 | 1 | 1 | 0 | 1 | 1 | 1 | 1 | 1 | 1 |
| *Mesosella simiola* Bates | *M.sim* | 0 | 1 | 1 | 0 | 0 | 0 | 0 | 0 | 0 | 0 | 0 | 0 | 1 | 0 |

**S2 Table** (continued 4)

| **Scientific name** | **Short name** | **Ito** | **Ogawa** | **Satomi** | **Mashiko** | **Odawara** | **Komaba** | **Koukyo** | **Akasaka** | **FFPRI** | **Fujisawa** | **Takao** | **MeijiJ** | **Mitake** | **Tzoo** |
| --- | --- | --- | --- | --- | --- | --- | --- | --- | --- | --- | --- | --- | --- | --- | --- |
| *Miaenia* (*Miaenia*) *tonsa* (Bates) | *M.ton* | 0 | 0 | 0 | 1 | 0 | 1 | 1 | 1 | 0 | 1 | 1 | 0 | 1 | 1 |
| *Miccolamia* (*Isomiccolamia*) *verrucosa* Bates | *M.ver* | 0 | 0 | 0 | 0 | 0 | 0 | 0 | 0 | 0 | 0 | 1 | 0 | 0 | 0 |
| *Miccolamia* (*Miccolamia*) *cleroides* Bates | *M.cle* | 0 | 0 | 1 | 0 | 0 | 0 | 0 | 0 | 0 | 0 | 1 | 0 | 1 | 0 |
| *Microlera ptinoides* Bates | *M.pti* | 0 | 0 | 0 | 0 | 0 | 0 | 1 | 0 | 1 | 0 | 1 | 1 | 1 | 0 |
| *Mimectatina divaricata divaricata* (Bates) | *M.div* | 0 | 0 | 0 | 0 | 0 | 0 | 0 | 0 | 1 | 0 | 1 | 0 | 0 | 0 |
| *Mimectatina meridiana ohirai* Breuning & Villiers | *M.mer* | 1 | 0 | 0 | 0 | 0 | 0 | 0 | 0 | 0 | 0 | 0 | 0 | 0 | 0 |
| *Molorchus* (*Molorchus*) *gracilis* Hayashi | *M.gra2* | 0 | 0 | 0 | 0 | 0 | 0 | 0 | 0 | 1 | 0 | 1 | 0 | 0 | 0 |
| *Molorchus* (*Molorchus*) *kobotokensis* K. Ohbayashi | *M.kob* | 0 | 0 | 0 | 0 | 0 | 0 | 0 | 0 | 0 | 0 | 1 | 0 | 0 | 0 |
| *Molorchus* (*Molorchus*) *kojimai* (Matsushita) | *M.koj* | 0 | 0 | 0 | 0 | 0 | 0 | 0 | 0 | 1 | 1 | 1 | 1 | 1 | 1 |
| *Monochamus* (*Monochamus*) *alternatus endai* Makihara | *M.alt* | 1 | 0 | 0 | 1 | 0 | 0 | 0 | 0 | 1 | 0 | 1 | 0 | 0 | 1 |
| *Monochamus* (*Monochamus*) *grandis* Waterhouse | *M.gra1* | 1 | 0 | 0 | 0 | 0 | 0 | 0 | 0 | 1 | 0 | 1 | 0 | 1 | 0 |
| *Monochamus* (*Monochamus*) *subfasciatus subfasciatus* Bates | *M.sub* | 1 | 1 | 0 | 1 | 0 | 0 | 0 | 0 | 1 | 1 | 1 | 1 | 1 | 1 |
| *Nanohammus rufescens* Bates | *N.ruf2* | 0 | 1 | 0 | 0 | 0 | 0 | 0 | 0 | 0 | 0 | 0 | 0 | 0 | 0 |
| *Necydalis* (*Eonecydalis*) *niimurai* Hayashi | *N.nii* | 0 | 0 | 0 | 0 | 0 | 0 | 0 | 0 | 0 | 0 | 0 | 0 | 1 | 0 |
| *Neocerambyx raddei* Blessig | *N.rad* | 0 | 0 | 0 | 1 | 0 | 0 | 0 | 1 | 1 | 1 | 1 | 0 | 0 | 1 |
| *Niphona* (*Niphona*) *furcata* (Bates) | *N.fur* | 1 | 0 | 0 | 1 | 0 | 0 | 0 | 0 | 1 | 1 | 1 | 0 | 0 | 1 |
| *Nothorhina punctata* (Fabricius) | *N.pun* | 0 | 0 | 0 | 0 | 0 | 1 | 1 | 0 | 0 | 0 | 0 | 1 | 0 | 1 |
| *Nupserha marginella marginella* (Bates) | *N.mar* | 0 | 1 | 1 | 0 | 1 | 0 | 0 | 0 | 1 | 0 | 1 | 0 | 1 | 1 |
| *Nysina rufescens rufescens* (Pic) | *N.ruf1* | 1 | 0 | 0 | 0 | 0 | 0 | 0 | 0 | 0 | 0 | 0 | 0 | 0 | 0 |
| *Oberea* (*Oberea*) *gracillima gracillima* Pascoe | *O.gra* | 0 | 0 | 0 | 0 | 0 | 0 | 0 | 0 | 0 | 0 | 1 | 0 | 0 | 0 |
| *Oberea* (*Oberea*) *hebescens* Bates | *O.heb* | 1 | 0 | 0 | 0 | 1 | 0 | 0 | 0 | 1 | 0 | 1 | 0 | 1 | 1 |
| *Oberea* (*Oberea*) *infranigrescens* Breuning | *O.inf* | 0 | 1 | 1 | 1 | 1 | 0 | 0 | 0 | 0 | 0 | 1 | 0 | 1 | 1 |

**S2 Table** (continued 5)

| **Scientific name** | **Short name** | **Ito** | **Ogawa** | **Satomi** | **Mashiko** | **Odawara** | **Komaba** | **Koukyo** | **Akasaka** | **FFPRI** | **Fujisawa** | **Takao** | **MeijiJ** | **Mitake** | **Tzoo** |
| --- | --- | --- | --- | --- | --- | --- | --- | --- | --- | --- | --- | --- | --- | --- | --- |
| *Oberea* (*Oberea*) *japonica* (Thunberg) | *O.jap* | 0 | 0 | 0 | 0 | 1 | 1 | 0 | 1 | 1 | 0 | 1 | 0 | 1 | 1 |
| *Oberea* (*Oberea*) *mixta* Bates | *O.mix* | 0 | 0 | 0 | 0 | 0 | 0 | 0 | 0 | 0 | 0 | 1 | 0 | 0 | 0 |
| *Oberea* (*Oberea*) *shirahatai* K. Ohbayashi | *O.shi* | 0 | 0 | 0 | 0 | 0 | 0 | 0 | 0 | 0 | 0 | 0 | 0 | 0 | 1 |
| *Oberea* (*Oberea*) *sobosana* K. Ohbayashi | *O.sob* | 0 | 0 | 0 | 0 | 0 | 0 | 0 | 0 | 0 | 0 | 0 | 0 | 1 | 0 |
| *Obrium nakanei* K. Ohbayashi | *O.nak* | 0 | 0 | 0 | 0 | 0 | 0 | 0 | 0 | 0 | 0 | 1 | 0 | 0 | 0 |
| *Ostedes* (*Ostedes*) *sapporensis* (Matsushita) | *O.sap* | 0 | 0 | 0 | 0 | 0 | 0 | 0 | 0 | 0 | 0 | 1 | 0 | 0 | 0 |
| *Pachytodes cometes* (Bates) | *P.com* | 0 | 0 | 0 | 0 | 0 | 0 | 0 | 0 | 0 | 0 | 0 | 0 | 1 | 0 |
| *Palimna liturata liturata* (Bates) | *P.lit* | 1 | 1 | 0 | 0 | 0 | 0 | 0 | 0 | 0 | 0 | 1 | 0 | 0 | 0 |
| *Paraclytus excultus* Bates | *P.exc* | 0 | 1 | 0 | 0 | 0 | 0 | 0 | 0 | 1 | 0 | 1 | 0 | 1 | 0 |
| *Paragaurotes doris doris* (Bates) | *P.dor* | 0 | 1 | 1 | 0 | 1 | 0 | 0 | 0 | 1 | 0 | 0 | 0 | 1 | 0 |
| *Paraglenea fortunei* (Saunders) | *P.for* | 1 | 0 | 0 | 0 | 1 | 1 | 0 | 0 | 1 | 0 | 0 | 0 | 1 | 0 |
| *Paramenesia kasugensis* (Seki & Kobayashi) | *P.kas* | 0 | 1 | 0 | 0 | 0 | 0 | 0 | 0 | 0 | 0 | 0 | 0 | 0 | 0 |
| *Paranaspia anaspidoides* (Bates) | *P.ana* | 0 | 0 | 0 | 0 | 1 | 1 | 1 | 0 | 1 | 1 | 1 | 1 | 0 | 1 |
| *Parastrangalis lesnei* (Pic) | *P.les* | 0 | 0 | 0 | 0 | 0 | 0 | 0 | 0 | 0 | 0 | 0 | 0 | 1 | 0 |
| *Parastrangalis nymphula* (Bates) | *P.nym* | 0 | 1 | 1 | 0 | 1 | 0 | 0 | 0 | 1 | 0 | 1 | 0 | 1 | 0 |
| *Parastrangalis tenuicornis* (Motschulsky) | *P.ten* | 0 | 1 | 1 | 0 | 1 | 0 | 0 | 0 | 0 | 0 | 1 | 0 | 1 | 0 |
| *Pareutetrapha eximia* (Bates) | *P.exi* | 0 | 0 | 0 | 0 | 0 | 0 | 0 | 0 | 1 | 0 | 1 | 0 | 0 | 0 |
| *Pareutetrapha simulans* (Bates) | *P.sim1* | 1 | 1 | 1 | 0 | 0 | 0 | 0 | 0 | 0 | 0 | 1 | 0 | 1 | 0 |
| *Pedostrangalia* (*Neosphenalia*) *femoralis* (Motschulsky) | *P.fem* | 0 | 0 | 0 | 0 | 0 | 0 | 0 | 0 | 0 | 0 | 1 | 0 | 0 | 0 |
| *Phymatodes* (*Paraphymatodes*) *albicinctus* Bates | *P.alb* | 0 | 1 | 1 | 0 | 0 | 0 | 0 | 0 | 0 | 0 | 1 | 0 | 0 | 0 |
| *Phymatodes* (*Phymatodes*) *testaceus* (Linnaeus) | *P.tes* | 0 | 1 | 0 | 0 | 0 | 0 | 0 | 0 | 1 | 0 | 1 | 0 | 0 | 1 |
| *Phymatodes* (*Poecilium*) *maaki viarius* Danilevsky | *P.maa* | 0 | 1 | 1 | 0 | 0 | 0 | 0 | 0 | 1 | 0 | 1 | 0 | 0 | 0 |
| *Phymatodes* (*Poecilium*) *quadrimaculatus* Gressitt | *P.qua* | 0 | 0 | 0 | 0 | 0 | 0 | 0 | 0 | 0 | 0 | 1 | 0 | 0 | 0 |
| *Phytoecia* (*Phytoecia*) *coeruleomicans* Breuning | *P.coe* | 0 | 0 | 0 | 0 | 0 | 0 | 0 | 0 | 0 | 0 | 1 | 0 | 0 | 0 |

**S2 Table** (continued 6)

| **Scientific name** | **Short name** | **Ito** | **Ogawa** | **Satomi** | **Mashiko** | **Odawara** | **Komaba** | **Koukyo** | **Akasaka** | **FFPRI** | **Fujisawa** | **Takao** | **MeijiJ** | **Mitake** | **Tzoo** |
| --- | --- | --- | --- | --- | --- | --- | --- | --- | --- | --- | --- | --- | --- | --- | --- |
| *Phytoecia* (*Phytoecia*) *rufiventris* Gautier | *P.ruf* | 0 | 1 | 1 | 0 | 0 | 1 | 0 | 0 | 0 | 0 | 1 | 0 | 0 | 1 |
| *Pidonia* (*Cryptopidonia*) *amentata amentata* (Bates) | *P.ame* | 0 | 1 | 1 | 0 | 0 | 0 | 0 | 0 | 1 | 0 | 1 | 0 | 0 | 0 |
| *Pidonia* (*Cryptopidonia*) *lyra* Kuboki & Suzuki | *P.lyr* | 0 | 0 | 0 | 0 | 0 | 0 | 0 | 0 | 0 | 0 | 1 | 0 | 0 | 0 |
| *Pidonia* (*Cryptopidonia*) *simillima* K. Ohbayashi & Hayashi | *P.sim2* | 0 | 1 | 1 | 0 | 0 | 0 | 0 | 0 | 1 | 0 | 0 | 0 | 1 | 0 |
| *Pidonia* (*Mumon*) *aegrota aegrota* (Bates) | *P.aeg* | 0 | 1 | 1 | 0 | 0 | 0 | 0 | 0 | 0 | 0 | 0 | 0 | 1 | 0 |
| *Pidonia* (*Omphalodera*) *puziloi* (Solsky) | *P.puz* | 0 | 1 | 1 | 0 | 1 | 0 | 0 | 0 | 1 | 0 | 1 | 0 | 1 | 0 |
| *Pidonia* (*Pseudopidonia*) *chairo* Tamanuki | *P.cha* | 0 | 1 | 0 | 0 | 0 | 0 | 0 | 0 | 0 | 0 | 0 | 0 | 0 | 0 |
| *Pidonia* (*Pseudopidonia*) *discoidalis* Pic | *P.dis* | 0 | 1 | 1 | 0 | 0 | 0 | 0 | 0 | 0 | 0 | 0 | 0 | 0 | 0 |
| *Pidonia* (*Pseudopidonia*) *grallatrix* (Bates) | *P.gra2* | 0 | 1 | 0 | 0 | 0 | 0 | 0 | 0 | 1 | 0 | 1 | 0 | 1 | 0 |
| *Pidonia* (*Pseudopidonia*) *signifera* (Bates) | *P.sig* | 0 | 1 | 1 | 0 | 1 | 0 | 0 | 0 | 0 | 0 | 0 | 0 | 1 | 0 |
| *Plagionotus christophi* (Kraatz) | *P.chi* | 0 | 1 | 0 | 0 | 0 | 0 | 0 | 0 | 0 | 0 | 1 | 0 | 0 | 0 |
| *Pogonocherus* (*Pogonocherus*) *dimidiatus* Blessig | *P.dim* | 0 | 1 | 1 | 0 | 0 | 0 | 0 | 0 | 1 | 0 | 1 | 0 | 0 | 0 |
| *Praolia citrinipes citrinipes* Bates | *P.cit* | 0 | 0 | 0 | 0 | 1 | 0 | 0 | 0 | 1 | 0 | 1 | 0 | 1 | 0 |
| *Prionus insularis insularis* Motschulsky (TypeA) | *P.ins1* | 1 | 0 | 0 | 0 | 0 | 0 | 0 | 0 | 0 | 0 | 0 | 0 | 0 | 0 |
| *Prionus insularis insularis* Motschulsky (TypeB) | *P.ins2* | 0 | 1 | 1 | 1 | 1 | 0 | 1 | 0 | 1 | 0 | 1 | 1 | 1 | 1 |
| *Prionus sejunctus* Hayashi | *P.sej* | 0 | 0 | 0 | 0 | 0 | 0 | 0 | 0 | 1 | 0 | 1 | 0 | 1 | 1 |
| *Psacothea hilaris hilaris* (Pascoe) | *P.hil* | 1 | 0 | 0 | 0 | 0 | 1 | 1 | 0 | 1 | 1 | 1 | 1 | 1 | 1 |
| *Psephactus remiger remiger* Harold | *P.rem* | 1 | 1 | 1 | 0 | 0 | 0 | 0 | 0 | 1 | 0 | 1 | 0 | 1 | 0 |
| *Pseudaeolesthes chrysothrix chrysothrix* (Bates) | *P.chr2* | 1 | 0 | 0 | 0 | 0 | 1 | 1 | 0 | 1 | 1 | 1 | 1 | 0 | 1 |
| *Pseudalosterna misella* (Bates) | *P.mis* | 0 | 1 | 1 | 0 | 0 | 0 | 0 | 0 | 0 | 0 | 0 | 0 | 1 | 0 |
| *Pseudocalamobius japonicus* (Bates) | *P.jap* | 0 | 1 | 1 | 0 | 0 | 0 | 0 | 0 | 0 | 0 | 1 | 0 | 1 | 0 |
| *Pterolophia* (*Ale*) *jugosa jugosa* (Bates) | *P.jug* | 0 | 1 | 1 | 0 | 0 | 0 | 0 | 0 | 1 | 0 | 1 | 0 | 1 | 1 |
| *Pterolophia* (*Hylobrotus*) *annulata* (Chevrolat) | *P.ann* | 1 | 0 | 0 | 1 | 0 | 0 | 1 | 0 | 1 | 1 | 1 | 0 | 0 | 0 |
| *Pterolophia* (*Pterolophia*) *angusta angusta* (Bates) | *P.ang* | 0 | 1 | 1 | 0 | 0 | 0 | 0 | 0 | 1 | 0 | 1 | 0 | 0 | 1 |
| *Pterolophia* (*Pterolophia*) *castaneivora* K. Ohbayashi & Hayashi | *P.cas* | 0 | 1 | 0 | 0 | 0 | 0 | 0 | 0 | 0 | 0 | 1 | 0 | 1 | 1 |

**S2 Table** (continued 7)

| **Scientific name** | **Short name** | **Ito** | **Ogawa** | **Satomi** | **Mashiko** | **Odawara** | **Komaba** | **Koukyo** | **Akasaka** | **FFPRI** | **Fujisawa** | **Takao** | **MeijiJ** | **Mitake** | **Tzoo** |
| --- | --- | --- | --- | --- | --- | --- | --- | --- | --- | --- | --- | --- | --- | --- | --- |
| *Pterolophia* (*Pterolophia*) *caudata caudata* (Bates) | *P.cau* | 1 | 1 | 1 | 1 | 1 | 0 | 1 | 0 | 1 | 0 | 1 | 1 | 1 | 1 |
| *Pterolophia* (*Pterolophia*) *granulata* (Motschulsky) | *P.gra1* | 1 | 1 | 1 | 1 | 1 | 1 | 1 | 0 | 1 | 0 | 1 | 1 | 1 | 1 |
| *Pterolophia* (*Pterolophia*) *leiopodina* (Bates) | *P.lei* | 1 | 1 | 0 | 0 | 0 | 0 | 0 | 0 | 0 | 0 | 1 | 0 | 0 | 0 |
| *Pterolophia* (*Pterolophia*) *tsurugiana* (Matsushita) | *P.tsu* | 0 | 1 | 1 | 0 | 1 | 0 | 0 | 0 | 0 | 0 | 1 | 0 | 1 | 0 |
| *Pterolophia* (*Pterolophia*) *zonata* (Bates) | *P.zon* | 1 | 1 | 1 | 1 | 1 | 1 | 1 | 1 | 1 | 1 | 1 | 1 | 0 | 1 |
| *Purpuricenus spectabilis* Motschulsky | *P.spe* | 0 | 0 | 0 | 0 | 1 | 0 | 0 | 0 | 1 | 0 | 1 | 0 | 1 | 0 |
| *Purpuricenus temminckii temminckii* Guerin-Meneville | *P.tem* | 1 | 0 | 0 | 0 | 0 | 0 | 0 | 0 | 1 | 1 | 1 | 0 | 1 | 1 |
| *Pyrestes munekuro* Fujimura | *P.mun* | 0 | 0 | 0 | 0 | 0 | 0 | 0 | 0 | 0 | 0 | 1 | 0 | 1 | 0 |
| *Rhagium* (*Rhagium*) *femorale* N. Ohbayashi | *R.fem* | 0 | 0 | 0 | 0 | 0 | 0 | 0 | 0 | 1 | 0 | 1 | 0 | 0 | 0 |
| *Rhaphuma diminuta diminuta* (Bates) | *R.dim* | 0 | 1 | 0 | 0 | 0 | 1 | 1 | 0 | 1 | 1 | 1 | 1 | 0 | 1 |
| *Rhaphuma xenisca* (Bates) | *R.xen* | 0 | 1 | 1 | 1 | 1 | 0 | 0 | 0 | 1 | 0 | 1 | 0 | 1 | 1 |
| *Rhodopina lewisii lewisii* (Bates) | *R.lew* | 1 | 0 | 0 | 1 | 0 | 0 | 0 | 0 | 1 | 0 | 1 | 0 | 1 | 1 |
| *Rhopaloscelis maculata* Bates | *R.mac* | 0 | 1 | 0 | 0 | 1 | 0 | 0 | 0 | 1 | 0 | 1 | 0 | 1 | 0 |
| *Rhopaloscelis unifasciata* Blessig | *R.uni* | 1 | 0 | 1 | 0 | 0 | 0 | 0 | 0 | 1 | 0 | 1 | 0 | 1 | 1 |
| *Rondibilis* (*Rondibilis*) *saperdina* (Bates) | *R.sap* | 1 | 1 | 0 | 1 | 1 | 0 | 0 | 0 | 0 | 0 | 1 | 0 | 1 | 1 |
| *Rosalia* *batesi* Harold | *R.bat* | 1 | 0 | 0 | 0 | 0 | 0 | 0 | 0 | 1 | 0 | 1 | 0 | 1 | 1 |
| *Saperda* (*Lopezcolonia*) *tetrastigma* Bates | *S.tet* | 0 | 0 | 0 | 0 | 1 | 0 | 0 | 0 | 1 | 0 | 1 | 0 | 0 | 0 |
| *Schwarzerium* (*Schwarzerium*) *quadricolle* (Bates) | *S.qua1* | 0 | 0 | 0 | 0 | 0 | 0 | 0 | 0 | 1 | 1 | 1 | 0 | 1 | 1 |
| *Semanotus bifasciatus* (Motschulsky) | *S.bif* | 0 | 0 | 0 | 0 | 0 | 0 | 0 | 0 | 0 | 0 | 1 | 0 | 0 | 0 |
| *Semanotus japonicus* Lacordaire | *S.jap1* | 0 | 0 | 0 | 0 | 0 | 0 | 0 | 0 | 1 | 1 | 1 | 0 | 0 | 1 |
| *Sophronica obrioides* (Bates, 1873) | *S.obr* | 0 | 0 | 0 | 0 | 0 | 0 | 0 | 0 | 0 | 0 | 1 | 0 | 0 | 0 |
| *Spondylis buprestoides* (Linnaeus, 1758) | *S.bup* | 1 | 1 | 1 | 1 | 1 | 0 | 1 | 1 | 1 | 1 | 1 | 0 | 1 | 1 |
| *Stenhomalus (Stenhomalus) japonicus* (Pic, 1904) | *S.jap2* | 0 | 0 | 0 | 0 | 0 | 0 | 0 | 0 | 0 | 0 | 1 | 0 | 1 | 0 |
| *Stenhomalus* (*Stenhomalus*) *taiwanus taiwanus* Matsushita | *S.tai* | 0 | 0 | 0 | 0 | 0 | 0 | 1 | 0 | 0 | 0 | 1 | 0 | 1 | 0 |

**S2 Table** (continued 8)

| **Scientific name** | **Short name** | **Ito** | **Ogawa** | **Satomi** | **Mashiko** | **Odawara** | **Komaba** | **Koukyo** | **Akasaka** | **FFPRI** | **Fujisawa** | **Takao** | **MeijiJ** | **Mitake** | **Tzoo** |
| --- | --- | --- | --- | --- | --- | --- | --- | --- | --- | --- | --- | --- | --- | --- | --- |
| *Stenhomalus* (*Stenhomalus*) *takaosanus* K. Ohbayashi | *S.tak* | 0 | 1 | 0 | 0 | 0 | 0 | 0 | 0 | 0 | 0 | 1 | 0 | 0 | 0 |
| *Stenodryas clavigera clavigera* Bates | *S.cla* | 0 | 0 | 0 | 0 | 0 | 0 | 0 | 0 | 1 | 0 | 1 | 0 | 1 | 0 |
| *Stenygrinum quadrinotatum* Bates | *S.qua2* | 0 | 1 | 1 | 0 | 0 | 0 | 0 | 0 | 1 | 0 | 1 | 0 | 0 | 1 |
| *Stictoleptura* (*Aredolpona*) *dichroa* (Blanchard) | *S.dic* | 0 | 1 | 1 | 1 | 1 | 0 | 0 | 0 | 1 | 0 | 1 | 1 | 1 | 1 |
| *Stictoleptura* (*Pyrrholeptura*) *pyrrha* (Bates) | *S.pyr* | 0 | 0 | 0 | 0 | 0 | 0 | 0 | 0 | 0 | 0 | 0 | 0 | 1 | 0 |
| *Strangalia koyaensis* Matsushita | *S.koy* | 0 | 0 | 0 | 0 | 0 | 0 | 0 | 0 | 0 | 0 | 0 | 0 | 1 | 0 |
| *Sybra* (*Microzotale*) *kuri* K. Ohbayashi & Hayashi | *S.kur* | 0 | 0 | 0 | 0 | 0 | 0 | 0 | 0 | 0 | 0 | 1 | 0 | 0 | 0 |
| *Sybra* (*Sybra*) *flavomaculata* Breuning | *S.fla* | 0 | 0 | 0 | 0 | 0 | 0 | 0 | 0 | 0 | 0 | 1 | 0 | 0 | 0 |
| *Sybra* (*Sybrodiboma*) *subfasciata subfasciata* Bates | *S.sub* | 0 | 1 | 1 | 0 | 1 | 0 | 0 | 0 | 1 | 0 | 1 | 0 | 1 | 0 |
| *Thyestilla gebleri* (Faldermann) | *T.geb* | 0 | 0 | 0 | 0 | 0 | 0 | 0 | 0 | 0 | 0 | 1 | 0 | 0 | 0 |
| *Toxotinus reinii* (Heyden) | *T.rei* | 0 | 1 | 1 | 0 | 1 | 0 | 0 | 0 | 1 | 0 | 1 | 0 | 1 | 0 |
| *Trichoferus campestris* (Faldermann) | *T.cam* | 0 | 0 | 0 | 0 | 0 | 0 | 0 | 0 | 0 | 0 | 0 | 0 | 0 | 1 |
| *Uraecha bimaculata bimaculata* Thomson | *U.bim* | 1 | 1 | 1 | 1 | 1 | 0 | 1 | 1 | 1 | 1 | 1 | 1 | 1 | 1 |
| *Xenicotela pardalina* (Bates) | *X.par* | 1 | 1 | 1 | 1 | 0 | 0 | 0 | 0 | 1 | 0 | 1 | 0 | 1 | 0 |
| *Xylariopsis mimica* Bates | *X.mim* | 0 | 0 | 0 | 0 | 0 | 0 | 0 | 0 | 0 | 0 | 1 | 0 | 0 | 0 |
| *Xylotrechus* (*Ootora*) *villioni* (Villard) | *X.vil* | 0 | 0 | 0 | 0 | 0 | 0 | 0 | 0 | 0 | 0 | 1 | 0 | 0 | 0 |
| *Xylotrechus* (*Xyloclytus*) *chinensis chinensis* (Chevrolat) | *X.chi* | 0 | 0 | 0 | 0 | 0 | 1 | 1 | 0 | 1 | 1 | 1 | 0 | 0 | 0 |
| *Xylotrechus* (*Xylotrechus*) *cuneipennis* (Kraatz) | *X.cun* | 1 | 1 | 1 | 0 | 0 | 0 | 0 | 0 | 1 | 0 | 0 | 0 | 1 | 0 |
| *Xylotrechus* (*Xylotrechus*) *emaciatus* Bates | *X.ema* | 1 | 1 | 1 | 0 | 1 | 0 | 0 | 0 | 1 | 0 | 1 | 0 | 1 | 0 |
| *Xylotrechus* (*Xylotrechus*) *grayii grayii* (White) | *X.gra* | 0 | 1 | 1 | 0 | 0 | 0 | 0 | 0 | 0 | 0 | 0 | 0 | 0 | 0 |
| *Xylotrechus* (*Xylotrechus*) *pyrrhoderus pyrrhoderus* Bates | *X.pyr* | 0 | 0 | 0 | 0 | 0 | 0 | 0 | 0 | 1 | 1 | 1 | 0 | 0 | 1 |
| *Xylotrechus* (*Xylotrechus*) *rufilius rufilius* Bates | *X.ruf* | 0 | 0 | 0 | 0 | 0 | 0 | 0 | 0 | 1 | 0 | 0 | 1 | 0 | 1 |
| *Xystrocera globosa* (Olivier) | *X.glo* | 1 | 0 | 0 | 0 | 0 | 0 | 0 | 0 | 1 | 0 | 1 | 0 | 0 | 0 |
